# Hepatoprotective potential of *Malvaviscus arboreus* against carbon tetrachloride-induced liver injury in rats

**DOI:** 10.1371/journal.pone.0202362

**Published:** 2018-08-23

**Authors:** Omnia Hesham Abdelhafez, Michael Atef Fawzy, John Refaat Fahim, Samar Yehia Desoukey, Markus Krischke, Martin J. Mueller, Usama Ramadan Abdelmohsen

**Affiliations:** 1 Department of Pharmacognosy, Faculty of Pharmacy, Deraya University, New Minia, Egypt; 2 Department of Biochemistry, Faculty of Pharmacy, Minia University, Minia, Egypt; 3 Department of Pharmacognosy, Faculty of Pharmacy, Minia University, Minia, Egypt; 4 Julius-von-Sachs-Institute of Biosciences, Biocenter, Pharmaceutical Biology, University of Würzburg, Würzburg, Germany; 5 Department of Botany II, Julius-von-Sachs-Institute for Biological Sciences, University of Würzburg, Würzburg, Germany; College of Agricultural Sciences, UNITED STATES

## Abstract

*Malvaviscus arboreus* Cav. is a medicinal plant belonging to family Malvaceae with both ethnomedical and culinary value; however, its phytochemical and biological profiles have been scarcely studied. Accordingly, this work was designed to explore the chemical composition and the hepatoprotective potential of *M*. *arboreus* against carbon tetrachloride (CCl_4_)-induced hepatotoxicity. The total extract of the aerial parts and its derived fractions (petroleum ether, dichloromethane, ethyl acetate, and aqueous) were orally administered to rats for six consecutive days, followed by injection of CCl_4_ (1:1 v/v, in olive oil, 1.5 ml/kg, i.p.) on the next day. Results showed that the ethyl acetate and dichloromethane fractions significantly alleviated liver injury in rats as indicated by the reduced levels of alanine transaminase (ALT), aspartate transaminase (AST), alkaline phosphatase (ALP), total bilirubin (TB), and malondialdehyde (MDA), along with enhancement of the total antioxidant capacities of their livers, with the maximum effects were recorded by the ethyl acetate fraction. Moreover, the protective actions of both fractions were comparable to those of silymarin (100 mg/kg), and have been also substantiated by histopathological evaluations. On the other hand, liquid chromatography-high resolution electrospray ionization mass spectrometry (LC‒HR‒ESI‒MS) metabolomic profiling of the crude extract of *M*. *arboreus* aerial parts showed the presence of a variety of phytochemicals, mostly phenolics, whereas the detailed chemical analysis of the most active fraction (*i*.*e*. ethyl acetate) resulted in the isolation and identification of six compounds for the first time in the genus, comprising four phenolic acids; *β*-resorcylic, caffeic, protocatechuic, and 4-hydroxyphenylacetic acids, in addition to two flavonoids; trifolin and astragalin. Such phenolic principles, together with their probable synergistic antioxidant and liver-protecting properties, seem to contribute to the observed hepatoprotective potential of *M*. *arboreus*.

## Introduction

Liver is a very important dynamic organ responsible for maintaining most of the vital physiological functions of the human body. It performs multiple regulating roles in different metabolic, secretory, and elimination processes [1]. It also represents the key organ for metabolism and detoxification of xenobiotics, and therefore is prone to many detrimental injuries with concurrent impairment of its vital functions leading to several life-threatening disorders, such as hepatitis, cirrhosis, hepatic failure, and dreadfully hepatocellular carcinoma [2,3]. Liver injuries are primarily induced by numerous agents, including toxic chemicals, e.g., CCl_4_ and aflatoxins, alcohol, drugs, and viruses, in addition to the hazardous environmental pollutants [2,3]. Such hepatic injuries are generally associated with elevation of the serum levels of liver enzymes, hepatocellular necrosis, plasma membrane damage, and enhanced oxidative stress with a remarkable depletion of glutathione [4,5]. Worldwide, liver diseases have undoubtedly become one of the rapidly increasing health burdens with elevated mortality rates. Moreover, along with the awful patients' suffering, current treatment approaches, including drug therapy and liver transplantation, are limitedly efficacious and are also accompanied by several risky complications [6]. These concerns have therefore stimulated the search for other safe and effective drug alternatives, particularly of natural origin. In that context, medicinal plants and their bioactive secondary metabolites have received considerable attention owing to their tremendous potential for management and correction of various forms of hepatopathy [3,5].

*Malvaviscus arboreus* Cav., syn. *Hibiscus malvaviscus* L., is a perennial herb or shrub belongs to the mallow family, Malvaceae [7]. It is indigenous to South and Central America, South-eastern United States, and Mexico [7,8]. It was also introduced to several tropical and subtropical areas in Australia, Asia, and Africa [7]. This plant is usually referred to as "Sleeping Hibiscus" because of its tightly wrapped red petals that do not open in full. Besides, it is commonly known as Turk's cap, Turk's turban, ladies teardrop, and Wax mallow [8]. Different *Malvaviscus* plants, including *M*. *arboreus*, are both showy ornamentals and culinary herbs, especially for their aerial parts, which are commonly used for preparation of salads, herbal teas, and herbal dyes [7]. Moreover, they have been traditionally used for the management of multiple health disorders, including fever, diarrhea, wounds, sore throat, tonsillitis, bronchitis, gastritis, stomachache, dysentery, liver and gall bladder problems, hypertension, cystitis, and kidney diseases [7,9–12].

So far, a limited number of phytochemical investigations have been conducted on *Malvaviscus* species, particularly *M*. *arboreus*, where flavonoids, anthocyanins, phenolic acids, sterols, and fatty acids were preliminary described as the major constituents [7,11,13]. Some other reports have also addressed their antimicrobial, thrombolytic, anti-inflammatory, membrane stabilizing, cytotoxic, and antioxidant potential [14–16]; however, their hepatoprotective properties have not been touched yet. Accordingly, the present study was undertaken to investigate the possible protective effects of the crude extract and different fractions of *M*. *arboreus* against CCl_4_-induced hepatotoxicity in rats, along with investigating its phytochemical composition through LC‒HR‒ESI‒MS metabolomic profiling, followed by a more comprehensive analysis of the most active fraction in order to explore the chemical principles that might contribute to its liver-protecting potential.

## Materials and methods

### Plant material

Leaves and stems of *M*. *arboreus* were collected in March 2015 from plants cultivated in the campus of Minia University, Minia, Egypt. Authentication of the plant was established by Prof. Mahmoud Abdelhady Hassan, Professor of Horticulture, Faculty of Agriculture, Minia University. A voucher specimen (Mn-Ph-Cog-027) was kept in the herbarium of Pharmacognosy Department, Faculty of Pharmacy, Minia University, Minia, Egypt.

### Chemicals and reagents

Solvents used in this work, e.g., petroleum ether (pet. ether; B.p. 60–80°C), dichloromethane (DCM), ethyl acetate (EtOAc), methanol (MeOH), and ethanol (EtOH), were purchased from El-Nasr Company for Pharmaceuticals and Chemicals, Egypt, and were distilled before use. Solvents of high performance liquid chromatography (HPLC) grade, e.g., CH_3_CN and MeOH, were used for HPLC separations and purifications, and were obtained from SDFCL sd Fine-Chem Limited, India. Deuterated solvents (Sigma-Aldrich, Germany), including methanol (CD_3_OD) and dimethyl sulfoxide (DMSO-*d*_*6*_), were used for nuclear magnetic resonance (NMR) spectroscopic analyses. Column chromatography (CC) was performed using silica gel 60 (E. Merck, Darmstadt, Germany; 60–120 mesh) or sephadex LH–20 (0.25–0.1 mm, GE Healthcare, Sweden), whereas silica gel GF_254_ for TLC (El-Nasr Company for Pharmaceuticals and Chemicals, Egypt) was employed for vacuum liquid chromatography (VLC). Thin layer chromatography (TLC) analyses were carried out using pre-coated silica gel 60 GF_254_ plates (E. Merck, Darmstadt, Germany; 20 × 20 cm, 0.25 mm in thickness). Spots were visualized by spraying with 10% sulfuric acid in methanol followed by heating at 110°C [17]. Ammonia vapors and aluminum chloride reagent (5% in ethanol) were also used for detection of flavonoids on TLC [18], while ferric chloride reagent (1% in ethanol) was used for phenolic compounds [19]. UV analysis of flavonoids was performed according to Mabry et al. using sodium methoxide, sodium acetate, aluminum chloride, and hydrochloric acid [20]. All chemicals used for the preparation of different spraying and UV reagents were obtained from El-Nasr Company for Pharmaceuticals and Chemicals, Egypt. For the biological study, silymarin was used as the standard hepatoprotective drug and was purchased from Pharco Pharmaceutical Company, Egypt, while both carboxymethylcellulose (CMC) and CCl_4_ were obtained from El-Nasr Company for Pharmaceuticals and Chemicals, Egypt.

### Apparatus

Ultraviolet lamp (UVP, LLC, USA) was used for visualization of spots on thin layer chromatograms at 254 and/or 365 nm. UV spectra of different samples were acquired using a Spectronic^®^ Genesys^TM^ 2PC UV spectrophotometer (Shimazdu, Japan) as solutions in methanol as well as with different diagnostic UV shift and complexing reagents for flavonoids [20]. ^1^H (400 MHz) as well as ^13^C NMR (100 MHz) and distortionless enhancement by polarization transfer (DEPT-Q; 100 MHz) spectra were recorded on Bruker Avance 400 MHz instruments in DMSO-*d*_*6*_ and CD_3_OD. Chemical shift values (*δ*) were recorded in ppm units and coupling constants (*J*) in Hz. Solvent signals of DMSO-*d*_*6*_ (*δ*_H_ 2.5 ppm and *δ*_C_ 39.5 ppm) and CD_3_OD (*δ*_H_ 3.3 ppm and *δ*_C_ 49.0 ppm) were considered as the internal reference signals for calibration. Electrospray ionization mass spectrometry (ESI‒MS) spectra were obtained using a Synapt G2 HDMS QTOF (quadrupole time-of-flight)-mass spectrometer (Waters, Germany). HPLC separations and purifications were performed on KNAUER HPLC (smart line pump 1000, degasser, diode array detector) with UV Detector, using semi-prep RP-18 column (5 μm, 10 × 250 mm; Waters XBridge, Germany), while an analytical Gemini-NX RP-18 column (5 μm, 4.60 × 100 mm; Phenomenex, Germany) was used for analytical purposes.

### Extraction and fractionation of plant material

The air dried, powdered leaves and stems (5 kg) of *M*. *arboreus* were extracted by maceration with 95% EtOH at room temperature and concentrated under reduced pressure to a syrupy consistency. The concentrated ethanolic extract (400 g) was suspended in distilled water (900 ml) and successively extracted with pet. ether, DCM, and EtOAc [21,22]. The organic phase in each step was separately evaporated under reduced pressure to afford the corresponding fractions I (90.0 g), II (9.0 g) and III (17.0 g), respectively, while the remaining mother liquor was then concentrated to give the aqueous fraction (IV). All the resulting fractions were kept at 4°C for the biological and phytochemical investigations.

### Phytochemical screening

The total ethanolic extract of *M*. *arboreus* aerial parts was assessed for the presence of carbohydrates and/or glycosides, steroids, triterpenoids, saponins, cardenolides, flavonoids, anthocyanins, coumarins, alkaloids and/or nitrogenous compounds, quinones, and tannins. Phytochemical screening was carried out using both chemical methods and TLC according to the standard procedures described by Sofowora [23] and Trease and Evans [24]. All chemicals used for preparation of different reagents were acquired from El-Nasr Company for Pharmaceuticals and Chemicals, Egypt.

### Metabolomics analysis

The crude ethanolic extract of *M*. *arboreus* was subjected to metabolomic analysis using analytical techniques of LC‒HR‒ESI‒MS according to Abdelmohsen et al. [25]. Briefly, the total extract (1 mg/ml in MeOH) was uploaded on an Accela HPLC (Thermo Fisher Scientific, Bremen, Germany) combined with Accela UV/VIS and Exactive (Orbitrap) mass spectrometer from Thermo Fisher Scientific (Bremen, Germany). The mobile phase composed of purified water (A) and acetonitrile (B) with 0.1% formic acid in each solvent. The gradient elution started at a flow rate of 300 μL/min with 10% B linearly increased to 100% B within 30 min and remained isocratic for the next 5 min before linearly decreasing back to 10% B for the following 1 min. The mobile phase was then equilibrated for 9 min before the next injection. The mass range was set from *m/z* (mass-to-charge ratio) 100‒2000 for ESI‒MS using in-source CID (collision-induced dissociation) mechanism and *m/z* 50‒1000 for MS/MS using untargeted HCD (high energy collision dissociation). In MZmine 2.12, a framework for differential analysis of mass spectrometry data, the raw data were imported. Chromatogram deconvolution was then performed followed by peaks deisotoping. For chromatographic alignment and gap-filling, the retention time normalizer was applied. Excel macros were used to combine positive and negative ionization mode data files generated by MZmine. Peaks produced from the sample were extracted. The Excel macro was used to dereplicate each *m/z* ion peak with compounds in the customized database (using RT and *m/z* threshold of ±5 ppm), which provided details on the putative identities of all metabolites in the total extract. The macro was then utilized to identify the top 20 features (ranked by peak intensity) and the corresponding putative identities by creating a list for the extract. Twelve metabolites **(1–12)** were therefore identified by comparison with some online and in-house databases.

### Isolation and purification of compounds

Because of exhibiting the highest hepatoprotective potential among the tested samples, the ethyl acetate soluble fraction (III) was selected for further investigation of its phytoconstituents, which possibly contribute to these protective effects. A part of fraction III (16 g) was subjected to VLC fractionation on a silica gel column (6 × 30 cm, 90 g). Elution was performed using pet. ether–EtOAc gradient mixtures in the order of increasing polarities (30, 70, and 100%), then with EtOAc–MeOH (70:30), and finally with MeOH. The effluents were collected in fractions (100 ml each); each fraction was concentrated and monitored by TLC. Similar fractions were grouped together and concentrated under reduced pressure to provide five subfractions (III_1_–III_5_). Subfraction III_2_ (2.0 g) was further fractionated on a silica gel column using DCM–MeOH gradient mixtures to yield five subfractions (III_2_–F–1: III_2_–F–5), of which III_2_–F–4 was then chromatographed on a sephadex LH–20 column (80 × 6 cm, 80 g) using DCM–Methanol (1:1) to give III_2_–F–4a: III_2_–F–4d. The obtained subfraction III_2_–F–4c was finally subjected to HPLC purification on a semi-preparative Waters XBridge RP-18 column (5 μm, 10 × 250 mm, Germany) using H_2_O–CH_3_CN (95:5) for 5 min, followed by a linear gradient to 100% CH_3_CN within 55 min, and finally with a further isocratic condition of CH_3_CN for 5 min at a flow rate of 2 ml/min to afford compounds **13** (5.0 mg; retention time (*R*_t_) = 20.0 min), **14** (3.0 mg; *R*_t_ = 21.0 min), and **15** (3.5 mg; *R*_t_ = 24.0 min).

Likewise, subfraction III_4_ (5.0 g) was chromatographed on a silica gel column (70 × 1.5 cm) employing gradient elution with DCM–MeOH to yield four subfractions (III_4_–F–1: III_4_–F–4). HPLC purification of III_4_–F–1 on a semi-preparative Waters XBridge RP-18 column (5 μm, 10 × 250 mm, Germany) using H_2_O–MeOH–CH_3_CN (95:4:1) for 5 min, followed by a linear gradient to H_2_O–MeOH–CH_3_CN (10:65:25) within 60 min at a flow rate of 2 ml/min, resulted in the isolation of compound **16** (3.8 mg; *R*_t_ = 17.0 min). In the same way, silica gel CC of subfraction III_4_–F–3 using gradient elution with DCM–MeOH provided three subfractions. Compounds **17** (2.0 mg; *R*_t_ = 23.1 min) and **18** (4.5 mg; *R*_t_ = 24.7 min) were then obtained from III_4_–F–3–1 by HPLC purification on a semi-preparative Waters XBridge RP-18 column using H_2_O–MeOH (90:10) for 2 min, followed by a linear gradient to 100% MeOH within 60 min at a flow rate of 2 ml/min.

### Hepatoprotective activity

#### Experimental animals

This study was conducted on adult male albino rats (150‒180 g, each) in compliance with the guidelines for the care and use of laboratory animals of the National Institutes of Health [26], and was also approved by the Research Ethics Committee for Animal Experimentation, Department of Pharmacology and Toxicology, Faculty of Pharmacy, Minia University, Egypt (project code No. 2017:019). Rats were housed and bred under standardized conditions in the pre-clinical animal house. They were kept in mesh-bottomed stainless steel cages (six per cage), fed a standard diet, and allowed free access to drinking water. The animals were acclimatized to the environment for one week before commencement of the experiment. All conditions were also made to minimize animal suffering.

#### Experimental design

The hepatoprotective activities of the total extract and its derived fractions were studied using the CCl_4_-induced liver injury model in rats according to Somasundaram et al. [27]. After an adaptation period of seven days, rats were randomly divided into eight groups of six animals each. Treatments were then carried out according to the following group allotment:

Group 1: served as the normal control group, which received the vehicle (0.05% CMC) only.
Group 2: received CCl_4_ only.
Group 3: received the standard drug silymarin (100 mg/kg per day in 0.05% CMC, p.o.) for six consecutive days.
Groups 4–7: received the pet. ether, DCM, EtOAc, and aqueous fractions (300 mg/kg per day in 0.05% CMC, p.o.), respectively, for six consecutive days.
Group 8: received the total extract (300 mg/kg per day in 0.05% CMC, p.o.) for six consecutive days.

For induction of liver injury, groups 2–8 received CCl_4_ in olive oil (1:1 v/v, 1.5 ml/kg, i.p.) as a single dose on the 7^th^ day, in addition to their basic treatments. On day 8, all rats were sacrificed by cervical decapitation. Blood samples were then collected from each group to determine the serum levels of ALT, AST, ALP, and TB. Liver tissues were also obtained to assess the levels of MDA, TAC, and for histopathological examination. All samples were stored at –80°C until the assay.

#### Determination of liver functions

The collected blood samples were centrifuged at 3000 rpm for 10 min. The obtained clear serum was used for measuring the levels of ALT, AST, ALP, and TB using commercially available kits according to the standard procedures [27].

#### Determination of MDA content and TAC

Liver tissues were washed with normal saline to remove any blood or blood clots. A part from the liver of each rat was homogenized with five times its weight with 0.1 M potassium phosphate buffer (pH 7.4) and centrifuged. After removal of cell debris, the supernatant was collected to assess the levels of MDA and TAC following the standard procedures [27,28].

#### Histopathological studies

Livers were immediately excised from the sacrificed rats. Liver tissues were fixed in 10% neutral buffered formalin, embedded in paraffin, and finally sectioned at 4–5 μm using a microtome. After removal of paraffin, the prepared sections were stained with alum-hematoxylin and eosin and evaluated microscopically for histopathological changes [29]. Finally, their images were captured using a LEICA, DM1000 microscope with a digital camera (LEICA, EC3, Germany).

#### Statistical analysis

Data were expressed as mean ± S.E.M (*n* = 6). One-way analysis of variance (ANOVA) followed by Dunnett's test was applied. Graph Pad Prism 5 was used for statistical calculations (Graph pad Software, San Diego, California, USA). Results were regarded as significant at *p* values less than 0.05, 0.01, and 0.001.

## Results

### Phytochemical screening

Phytochemical qualitative analysis of the total ethanolic extract of *M*. *arboreus* aerial parts revealed the presence of carbohydrates and/or glycosides, steroids, triterpenoids, flavonoids, anthocyanins, and coumarins, whereas crystalline sublimates, cardenolides, saponins, alkaloids and/or nitrogenous compounds, quinones, and tannins were absent.

### Metabolomic analysis

**Fig 1:**
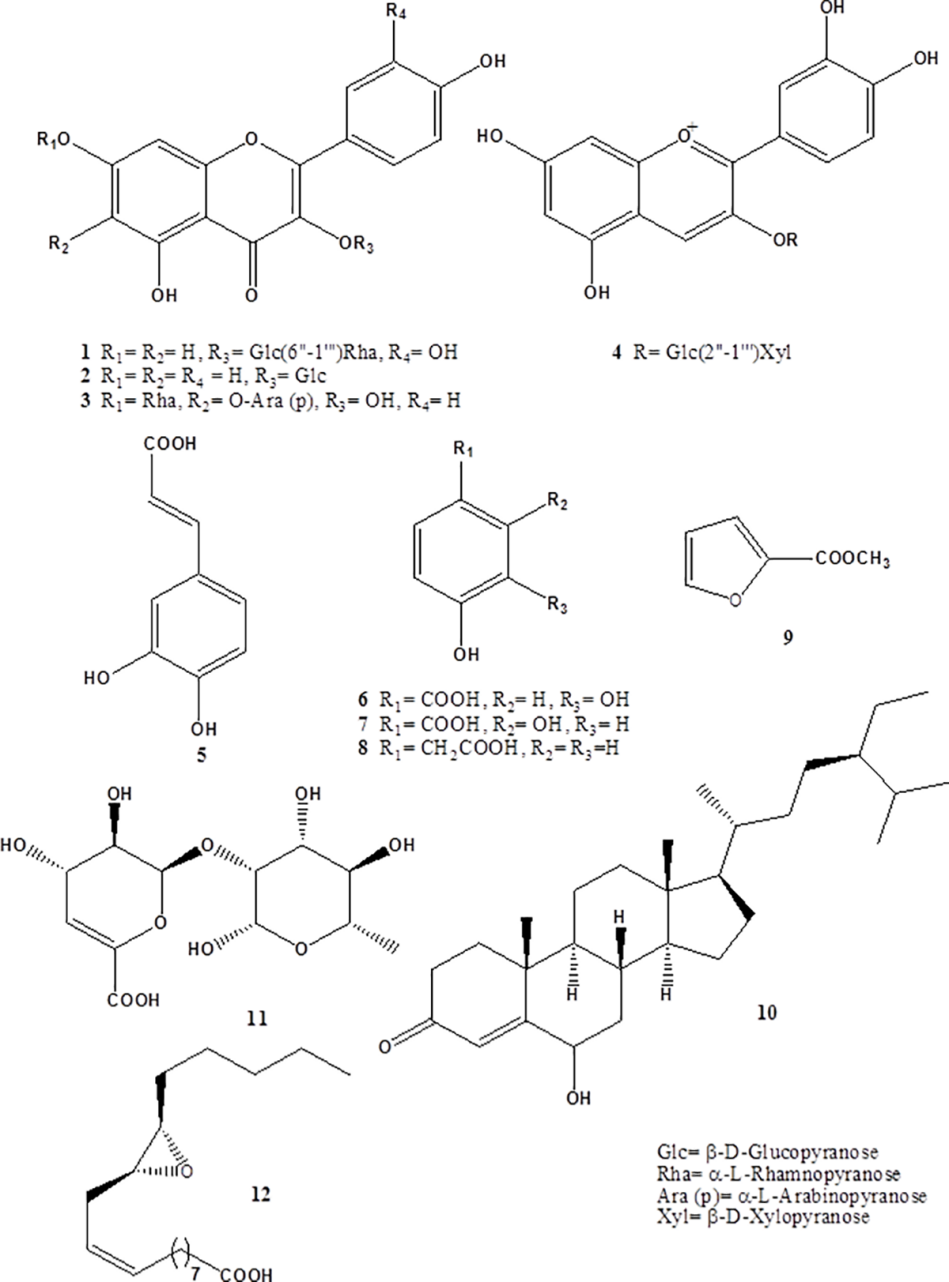
Structures of the dereplicated metabolites from *M*. *arboreus*.

Chemical profiling of the secondary metabolites of *M*. *arboreus* aerial parts, using LC‒HR‒ESI‒MS for dereplication purposes, has resulted in the characterization of a variety of constituents, among which phenolics such as flavonoids and phenolic acids predominated. The detected compounds (Fig 1) were identified by employing macros and algorithms that coupled MZmine with online and in-house databases (METLIN and DNP databases for plant natural products). From these databases, the mass ion peak at *m/z* 610.150 for the predicted molecular formula C_27_H_30_O_16_ was dereplicated as the flavonoidal glycoside rutin (**1**), which was previously detected in *M*. *arboreus* by HPLC analysis of its flowers [11], whereas that at *m/z* 448.100, corresponding to the suggested molecular formula C_21_H_20_O_11_, was dereplicated as astragalin (**2**), which was formerly reported from the related species *M*. *conzattii* Greenm. [13]. Likewise, a flavone glycoside with the molecular formula C_28_H_28_O_14_, was characterized as 4',5,6,7-tetrahydroxyflavone 6-*O*-*β*-arabinopyranoside 7-*O*-*α*-rhamnopyranoside (**3**) from the mass ion peak at *m/z* 565.155. This apigenin bioside was previously obtained from *Urena lobata* L. [30], a Malvaceous plant belonging to the tribe *Hibisceae*, while this is the first report for this glycoside in the genus *Malvaviscus*. Moreover, the mass ion peak at *m/z* 581.150, in agreement with the predicted molecular formula C_26_H_29_O_15_, was dereplicated as cyanidin 3-sambubioside (**4**), an anthocyanin earlier isolated from several *Hibiscus* species [31], whereas it is reported herein for the first time from *Malvaviscus* plants.

In addition to the above-mentioned flavonoidal molecules, metabolomic analysis of *M*. *arboreus* showed that the phenolic pool of this plant comprises a number of free phenolic acids, including derivatives of benzoic and hydroxycinnamic acids. In this context, the mass ion peak at *m/z* 180.043 for the suggested molecular formula C_9_H_8_O_4_ was identified as caffeic acid (**5**), whereas that at *m/z* 154.045, consistent with the molecular formula C_7_H_6_O_4_, was dereplicated as protocatechuic acid (**6**) and/or *β*‒resorcylic acid (**7**). The former was previously detected in *M*. *arboreus* flowers by HPLC analysis [11], while the latter has not been reported from the genus yet. Another related compound was also characterized as 4-hydroxyphenylacetic acid (**8**), for the first time from the genus, based on the mass ion peak at *m/z* 152.054 and in accordance with the molecular formula C_8_H_8_O_3_.

Aside from the characterized phenolic metabolites, a number of other phytoconstituents belonging to different structural types were also described; comprising steroids, furanoids, pyranoids, and long chain fatty acids. In this regard, the mass ion peak at *m/z* 126.039, corresponding to the suggested molecular formula C_6_H_6_O_3_, was dereplicated as 2-furancarboxylic acid methyl ester (**9**), while that at *m/z* 428.370 was dereplicated as 6-hydroxystigmast-4-en-3-one (**10**) with the molecular formula C_29_H_48_O_2_, which was obtained before from *Hibiscus esculentus* L., commonly known as okra [32]. Lepidiomic acid (**11**), a polyhydroxylated pyranoid previously identified from *H*. *esculentus* [33], was also characterized from the mass ion peak at *m/z* 322.090 in agreement with the predicted formula C_12_H_18_O_10_, whereas that at *m/z* 296.242 was dereplicated as the unsaturated epoxy fatty acid, vernolic acid (**12**), with the molecular formula C_18_H_32_O_3_, which was also formerly isolated from several *Hibiscus* species [34]. In light of these findings, it is worth mentioning that this is the first report for metabolites **3–12** from the genus *Malvaviscus*. Alongside their direct correlation with the potential bioactivities of *M*. *arboreus*, these phytochemical data concerning this rarely studied species are of appreciable chemotaxonomic value too.

### Structure elucidation of the isolated compounds

**Fig 2:**
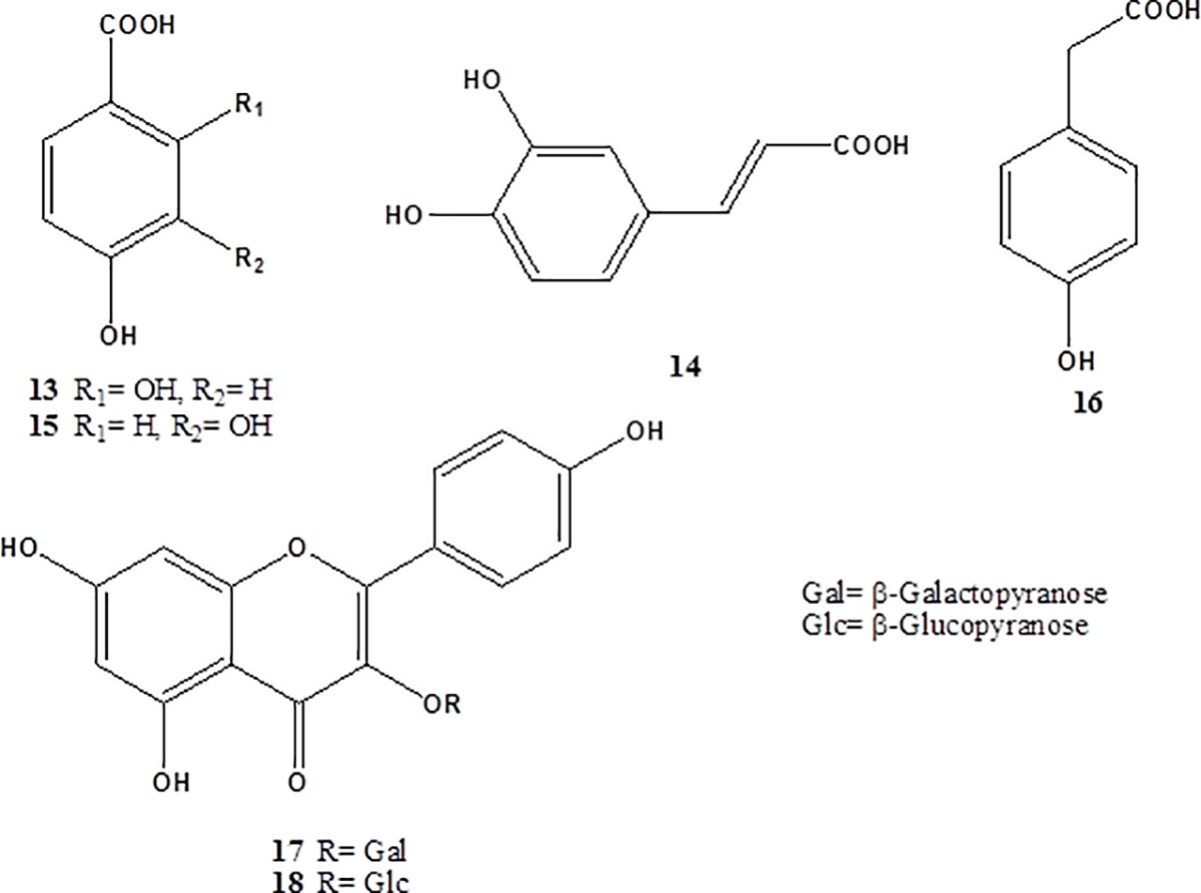
Structures of the isolated compounds from *M*. *arboreus*.

Based on their physicochemical and chromatographic properties, spectral analyses (UV, ESI‒MS, ^1^H and ^13^C NMR, and DEPT‒Q), as well as comparison with the literature and some authentic samples, the isolated compounds (Fig 2) were identified as *β*–resorcylic acid (*p*-hydroxysalicylic acid; **13**) [35], caffeic acid (**14**) [36], protocatechuic acid (**15**) [37], 4-hydroxyphenylacetic acid (**16**) [38], kaempferol 3-*O*-*β*-galactopyranoside (trifolin; **17**) [39], and kaempferol 3-*O*-*β*-glucopyranoside (astragalin; **18**) [21]. All the characterized metabolites (**13–18**) were isolated herein for the first time from the genus *Malvaviscus*.

### Effect of *M*. *arboreus* on serum biochemical parameters

**Fig 3:**
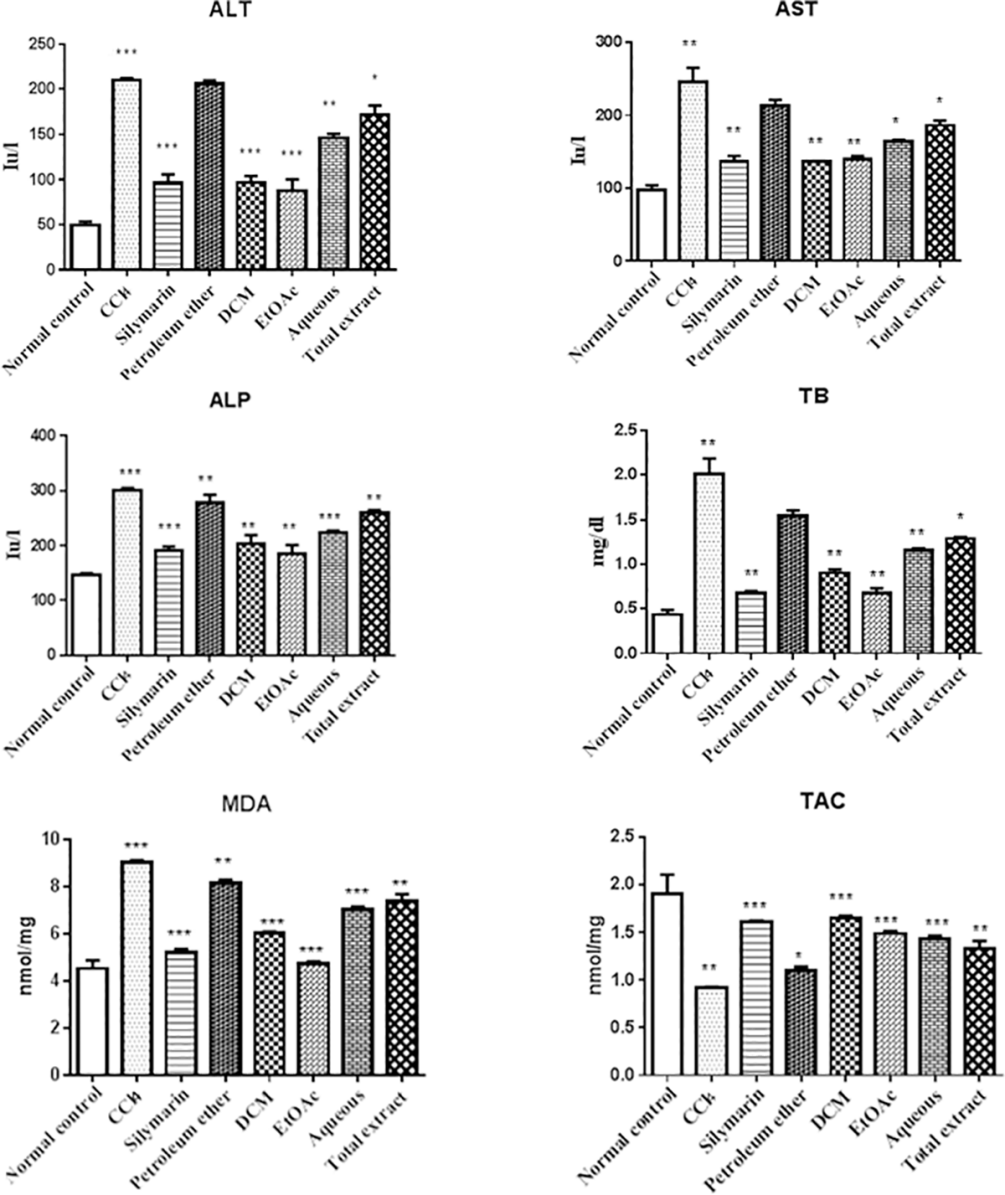
Effect of the total extract and various fractions of *M*. *arboreus* on different biochemical parameters in CCl_4_-intoxicated rats. Values are mean ± S.E.M (*n* = 6). *Statistically significant differences (*p* < 0.05, compared with the CCl_4_-treated group), **Statistically significant differences (*p* < 0.01, compared with the CCl_4_-treated group), ***Statistically significant differences (*p* < 0.001, compared with the CCl_4_-treated group).

As shown in Fig 3, the serum levels of ALT, AST, ALP, and TB were significantly (*p* < 0.01 or *p* < 0.001) increased after administration of CCl_4_ as compared to the normal control group, indicating an acute liver damage after CCl_4_ intoxication. In contrast, pretreatment of rats with the standard drug silymarin (100 mg/kg, p.o.) for six consecutive days significantly (*p* < 0.01 or *p* < 0.001) reduced the CCl_4_-induced elevation in the serum levels of the aforementioned parameters, implying to a substantial improvement of their liver functions. Except for the pet. ether fraction, the total extract and its derived fractions of *M*. *arboreus* significantly lowered the CCl_4_-elevated levels of ALT, AST, ALP, and TB, and amongst them, the EtOAc and DCM fractions exhibited the maximum effects, respectively, that were also comparable to those of silymarin. Therefore, pretreatment of rats with the EtOAc and DCM fractions for six consecutive days before CCl_4_ administration markedly decreased the extent of their liver injury.

### Effect of *M*. *arboreus* on MDA content and TAC

Administration of rats with CCl_4_ produced a significant (*P* < 0.001) increase in the MDA levels of liver tissues relative to the vehicle group, as shown in Fig 3. Besides, their livers showed significantly (*P* < 0.01) lower TAC as compared to the control group. Conversely, pretreatment with silymarin significantly (*P* < 0.001) reduced the elevated levels of MDA and normalized the TAC in CCl_4_-intoxicated rats. Likewise, the crude extract and different fractions of *M*. *arboreus* significantly (*p* < 0.01 or *p* < 0.001) lowered the increased MDA levels in rats' livers and also enhanced their TAC. In the same way, the EtOAc and DCM fractions were the most effective among the tested fractions, respectively, and their effects were also comparable to those of the standard drug silymarin. Moreover, the elevated MDA levels were nearly totally reversed in the EtOAc fraction-treated rats.

### Histopathological examination

**Fig 4:**
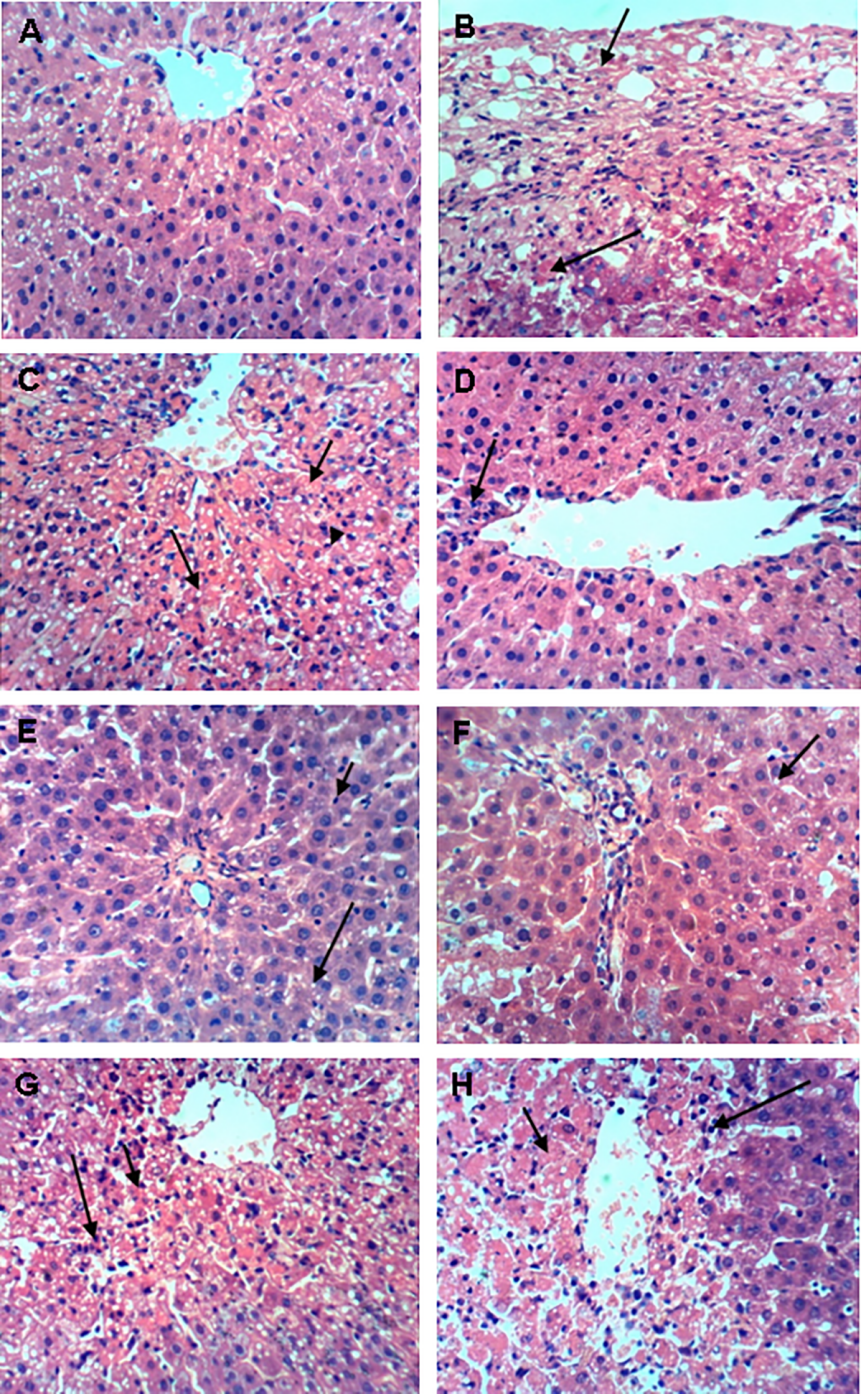
Histopathology of hepatic tissue sections (× 400). A: Normal control group, B: CCl_4_ model group, C: Silymarin, D: Pet. ether fraction, E: DCM fraction, F: EtOAc fraction, G: Aqueous fraction, H: Total extract.

The histopathological changes observed in the liver tissues of different groups are illustrated in Fig 4. Liver sections of the control group (Fig 4A) displayed normal well-preserved hepatocytes with prominent nucleus, nucleolus, uniform cytoplasm, and radial arrangement around the central vein, as well as well-defined sinusoids. In contrast, severe injuries were noticed in liver tissues of the CCl_4_-treated model group, as shown by the remarkable perihepatitis (severe inflammation of the hepatic capsule), widespread necrosis of sub-capsular hepatocytes, extensive infiltration of inflammatory cells, and fatty changes (Fig 4B). In comparison with the model group, pretreatment of CCl_4_-intoxicated rats with silymarin markedly alleviated their hepatocellular damage (Fig 4C) as reflected by the reduction of necrotic areas and inflammatory cell infiltration. In harmony with the previous biochemical analyses, the potential hepatoprotective effects of the EtOAc and DCM fractions of *M*. *arboreus* were also clearly evidenced by the observed histopathological findings. For the DCM fraction-treated rats, mild changes in Kupffer cells activation as well as mild fatty changes were detected, along with the presence of small vacuoles in the cytoplasm of hepatocytes (Fig 4E). Likewise, slight fatty changes of hepatocytes accompanied by mild changes in Kupffer cells activation were noticed in those pretreated with the EtOAc fraction (Fig 4F). Both fractions significantly decreased necrosis and ameliorated the hepatocellular hypertrophy. They also obviously reduced the number of degenerated hepatocytes and the extent of inflammatory cell infiltration. Moreover, a relatively intact central vein was distinguished with both fractions (Fig 4E and 4F). On the other hand, liver sections from both the total extract- and aqueous fraction-treated groups showed a moderate damage of liver's architecture. This was indicated by a moderate hepatocellular necrosis in addition to some inflammatory cells infiltration in those treated with the aqueous fraction, whereas moderate fatty changes accompanied with slight Kupffer cells infiltration were detected in the case of the total extract (Fig 4G and 4H). Concomitantly, liver sections of the pet. ether fraction-treated rats exhibited some foci of hepatic necrosis (Fig 4D).

## Discussion

CCl_4_-induced hepatoxicity is a commonly used model in testing the hepatoprotective properties of medicinal plants and their phytoconstituents against chemical liver injury, and is also a good representative for a number of liver disorders, such as fatty liver, fibrosis, and cirrhosis [40,41]. The hepatic damage caused by CCl_4_ is generally mediated by its bioactivation to the free radicals ^•^CCl_3_ and CCl_3_OO^•^, which have the capacity to initiate several injurious intracellular and extracellular events [42]. These active metabolites of CCl_4_ attack and destroy polyunsaturated fatty acids, especially those associated with phospholipids, resulting in lipid peroxidation in cellular and organelle membranes, which subsequently produces severe disturbances of calcium homeostasis and necrotic cell death due to the increase in the permeability of plasma membranes to calcium ions [5,43]. As a result, hepatocytes suffer from a marked disruption of cell integrity, followed by excessive leakage of transaminases into the blood, leading to a substantial rise in serum ALT and AST levels, which is a distinctive feature of hepatonecrosis [5]. Some tissue macromolecules like proteins can also be attacked by the generated free radicals as well as by certain products of lipid peroxidation processes [41]. Additionally, stimulation of Kuppfer cells, the resident macrophages in the liver, exacerbates liver inflammation either through oxidative stress or by tumor necrosis factor (TNF)-α release, which finally leads to further hepatocellular apoptosis [44].

In this work, the hepatoprotective potential of the total extract of *M*. *arboreus* aerial parts and its derived fractions were examined using the CCl_4_-induced liver injury in rats. Liver functions of the model CCl_4_-treated animals were seriously affected, as reflected by the remarkable elevation of their serum biomarkers in comparison with the normal control group, which is a primary feature of hepatic damage. According to Navarro and Senior [4], CCl_4_-induced liver injury can be described as hepatocellular (with elevated ALT levels), cholestatic (with increased levels of ALP and TB), or mixed (with elevated levels of ALT and ALP). Based on the obtained results, the current experimental model showed mixed hepatotoxicity since all the measured parameters were significantly affected. However, pretreatment of rats with the total extract and different fractions of *M*. *arboreus* has decreased the extent of their liver damage with variable degrees, where the highest protective effects were observed for the EtOAc and DCM fractions, respectively. The EtOAc and DCM fractions significantly prevented the CCl_4_-induced elevation of serum ALT by 76.1% and 70.5% and AST by 71.8% and 74.3%, respectively. They also reduced the increased levels of ALP by 75.1% and 62.8% as well as TB by 84.4% and 70.6%, respectively. Their protective actions were evidently comparable to those of the positive drug silymarin (100 mg/kg), which restored the levels of ALT, AST, ALP, and TB by 70.9, 73.8, 71.4, and 85.0%, respectively. Besides, both the EtOAc fraction and silymarin equally improved the raised levels of TB (84.4% vs. 85.0%). These biochemical findings were also concomitantly substantiated with the histopathological evidences as described before.

Biological systems involve a wide range of enzymatic (e.g., glutathione peroxidase, superoxide dismutase, and catalase) and non-enzymatic (e.g., ascorbate, tocopherols, carotenoids, bilirubin, and uric acid) antioxidants with tightly-controlled levels in order to keep their endogenous redox balance [45]. Oxidative stress on the other hand is developed as a result of the imbalance between the antioxidant and oxidant systems, with a tendency towards the latter. Free radicals generated on disturbance of the normal redox state could damagingly attack some biomolecules, such as lipids, proteins, and DNA, thus affecting cell membrane integrity via lipid peroxidation and could also initiate DNA mutations [22,46]. Furthermore, MDA is a major end product resulting from free radical attack on polyunsaturated fatty acids of biological membranes, and is usually used for monitoring lipid peroxidation. The accumulation of this aldehyde in excessive amounts, such as in CCl_4_-injured livers, implies to the inability of endogenous antioxidant systems to stop the production of further toxic radicals, leading to progressive peroxidation and subsequent hepatic tissue damage [47].

Likewise, total antioxidant capacity (TAC) is another early marker of oxidative stress that reflects the cumulative effect of all antioxidants found in a biological system [45]. Although the levels of endogenous antioxidant components can be estimated individually, they may not accurately show the total antioxidant power; therefore, the measurement of TAC provides the exact endogenous antioxidant status [45,48]. In the present study, the hepatocellular level of oxidative stress was enhanced by CCl_4_ intoxication, as shown by the elevated MDA content of liver tissues as well as the lowered TAC relative to the normal control group. Pretreatment of rats with the total extract and different fractions of *M*. *arboreus* has decreased the elevated liver MDA and enhanced TAC with varied degrees, with the maximum effects were noticed for the EtOAc and DCM fractions, respectively. The EtOAc and DCM fractions significantly diminished the CCl_4_-induced elevation of MDA levels by 95.6% and 66.6% (versus 85.1% for silymarin), and improved the hepatic TAC by 58.6% and 74.7% (versus 70.7% for silymarin), respectively.

Based on the obtained biochemical and histopathological findings, the current study demonstrated the potential hepatoprotective properties of *M*. *arboreus* against CCl_4_-induced hepatotoxicity in rats, particularly of its EtOAc and DCM fractions, which is also consistent with its folk use in the treatment of multiple gall bladder and liver ailments. In that context, the noticeable reduction of CCl_4_-elevated ALT and AST levels caused by both fractions suggests stabilization of plasma membranes as well as alleviation or repair of hepatocellular damage, whereas the concomitant diminishing of the raised ALP and bilirubin levels indicates the improvement of biliary dysfunction [49]. Besides, it was reported that the anti-inflammatory and antioxidant activities of medicinal plants might largely contribute to the possible mechanisms of their hepatoprotective potential [50]. In view of that, the observed protective effects of the EtOAc and DCM fractions may be partially mediated by their *in vivo* antioxidant properties, as reflected by the significant reduction of MDA levels and enhancement of TAC. Such antioxidant potential is mostly underlain by their phenolic principles, and is finally translated into an improvement of the endogenous scavenging of free radicals as well as the total antioxidant status, thus preserving the structural integrity of hepatocytes. As a result, and on account of the limited phytochemical data concerning *Malvaviscus* plants, the aerial parts of *M*. *arboreus* were subjected to a preliminary phytochemical screening, then LC‒HR‒ESI‒MS metabolomic profiling of their secondary metabolites, followed by a detailed phytochemical analysis of the EtOAc fraction, as the most active one, in order to dig out the different chemical principles that possibly contribute to its bioactivity. Results of both the phytochemical screening and metabolomic analysis of *M*. *arboreus* revealed its richness in phenolic compounds, such as flavonoids, anthocyanins, phenolic acids, and coumarins. In addition, metabolomic profiling of *M*. *arboreus* showed the presence of a variety of metabolites with well-known hepatoprotective properties. Furthermore, phytochemical investigation of the EtOAc fraction has resulted in the isolation and characterization of a number of phenolic acids, including *β*-resorcylic, caffeic, protocatechuic, and 4-hydroxyphenylacetic acids, in addition to two flavonoidal glycosides; trifolin and astragalin. Such plant phenolics, including flavonoids, anthocyanins, and phenolic acids, are well reputed for their ability to prevent xenobiotic-induced hepatotoxicity in experimental animals, mostly due to their antioxidant and free radical scavenging potential [46,51]. Amongst the characterized metabolites herein, astragalin, rutin, trifolin, cyanidin 3-sambubioside, caffeic acid, protocatechuic acid, and 4-hydroxyphenylacetic acid have been formerly reported to possess noteworthy anti-oxidative and liver-protecting activities [52–60]. Consequently, the notable hepatoprotective potential of *M*. *arboreus* could be partly related to the combined effects of these phytochemicals and/or their synergistic interactions.

## Conclusion

The present work revealed the hepatoprotective effects of the aerial parts of *M*. *arboreus* against CCl_4_-induced liver injury, particularly of their ethyl acetate and dichloromethane fractions, that were comparable to silymarin. In addition, metabolomic and phytochemical analyses of the plant showed its ability to biosynthesize and accumulate a variety of secondary metabolites, predominantly phenolics, which largely suggest their contribution to the witnessed protective actions of *M*. *arboreus*, either through their hepatotoxicity-alleviating effects and/or antioxidative traits. These findings might help widen the applications of this plant in future phytotherapy, and with regard to its reported edibility, dietary supplementation using *M*. *arboreus* could be hopefully considered to protect against liver damage. Further investigation of the cellular mechanisms and molecular aspects of the hepatoprotective potential of *M*. *arboreus* is therefore recommended in the near future.

## Supporting information

S1 Fig Total ion chromatogram of the total ethanolic extract of *M*. *arboreus* aerial parts. (TIF)

S2 Fig HPLC chromatogram of compound (13). (TIF)

S3 Fig ^1^H-NMR spectrum of compound (13) (CD_3_OD, 400 MHz). (TIF)

S4 Fig DEPT-Q spectrum of compound (13) (CD_3_OD, 100 MHz). (TIF)

S5 Fig HPLC chromatogram of compound (14). (TIF)

S6 Fig ^1^H-NMR spectrum of compound (14) (CD_3_OD, 400 MHz). (TIF)

S7 Fig Expanded ^1^H-NMR spectrum of compound (14) (CD_3_OD, 400 MHz). (TIF)

S8 Fig DEPT-Q spectrum of compound (14) (CD_3_OD, 100 MHz). (TIF)

S9 Fig Expanded DEPT-Q spectrum of compound (14) (CD_3_OD, 100 MHz). (TIF)

S10 Fig ESI-MS spectrum of compound (14). (TIF)

S11 Fig HPLC chromatogram of compound (15). (TIF)

S12 Fig ^1^H-NMR spectrum of compound (15) (DMSO-*d*_*6*_, 400 MHz). (TIF)

S13 Fig Expanded ^1^H-NMR spectrum of compound (15) (DMSO-*d*_*6*_, 400 MHz). (TIF)

S14 Fig ESI-MS spectrum of compound (15). (TIF)

S15 Fig HPLC chromatogram of compound (16). (TIF)

S16 Fig ^1^H-NMR spectrum of compound (16) (CD_3_OD, 400 MHz). (TIF)

S17 Fig Expanded ^1^H-NMR spectrum of compound (16) (CD_3_OD, 400 MHz). (TIF)

S18 Fig ESI-MS spectrum of compound (16). (TIF)

S19 Fig HPLC chromatogram of compound (17). (TIF)

S20 Fig ^1^H-NMR spectrum of compound (17) (DMSO-*d*_*6*_, 400 MHz). (TIF)

S21 Fig Expanded ^1^H-NMR spectrum of compound (17) (DMSO-*d*_*6*_, 400 MHz). (TIF)

S22 Fig HPLC chromatogram of compound (18). (TIF)

S23 Fig ^1^H-NMR spectrum of compound (18) (DMSO-*d*_*6*_, 400 MHz). (TIF)

S24 Fig ^13^C NMR spectrum of compound (18) (DMSO-*d*_*6*_, 100 MHz). (TIF)

S1 Table Effect of the total extract and various fractions of *M*. *arboreus* on different biochemical parameters in CCl_4_-intoxicated rats. (PDF)
