# Supplementary figures and images for: Hepatoprotective potential of Malvaviscus arboreus against carbon tetrachloride-induced liver injury in rats
Source: PLoS One. 2018 Aug 23;13(8):e0202362. doi: 10.1371/journal.pone.0202362 (PMC6107176; doi:10.1371/journal.pone.0202362)

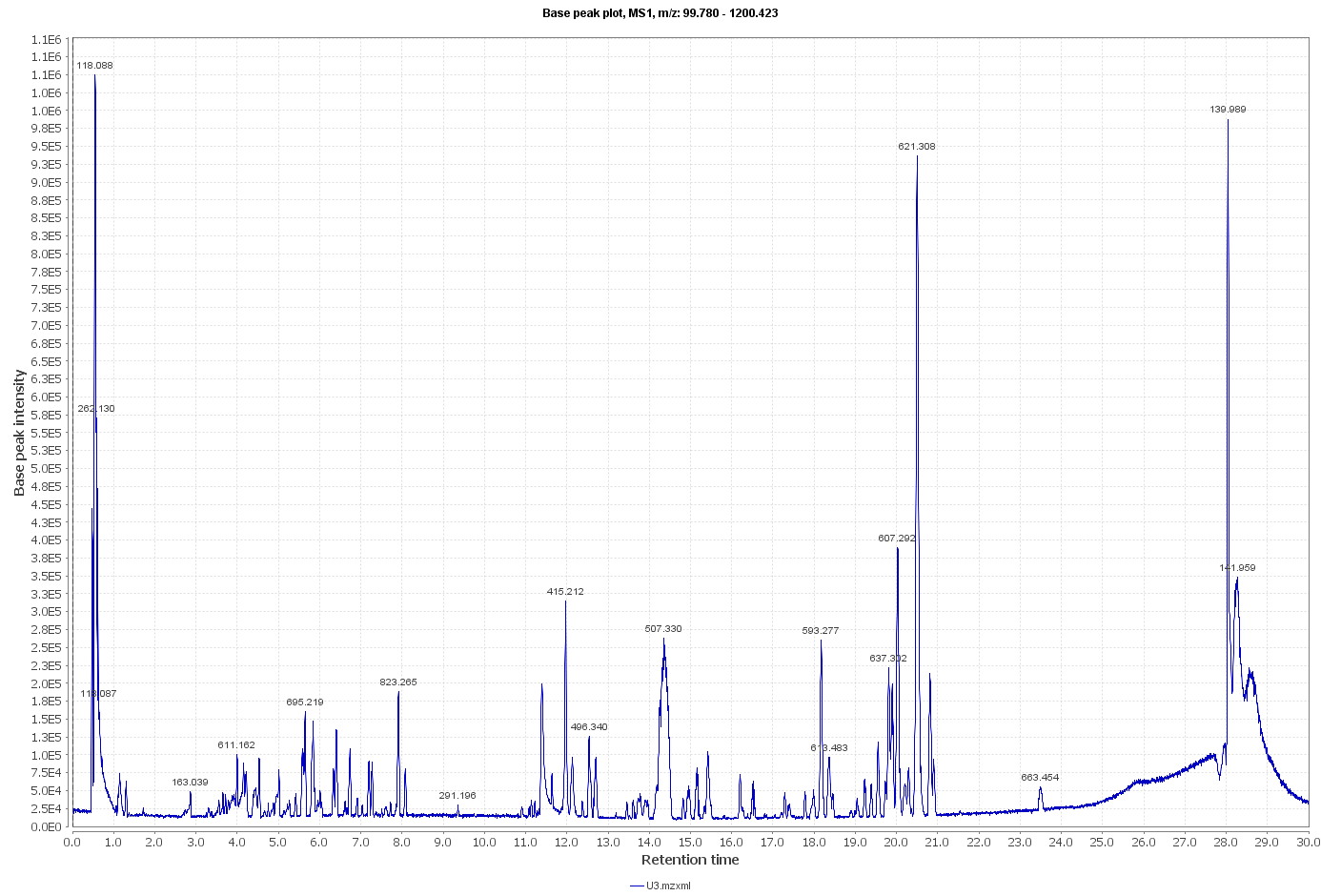

Supplement: S1 Fig — (TIF) [file pone.0202362.s001.tif]

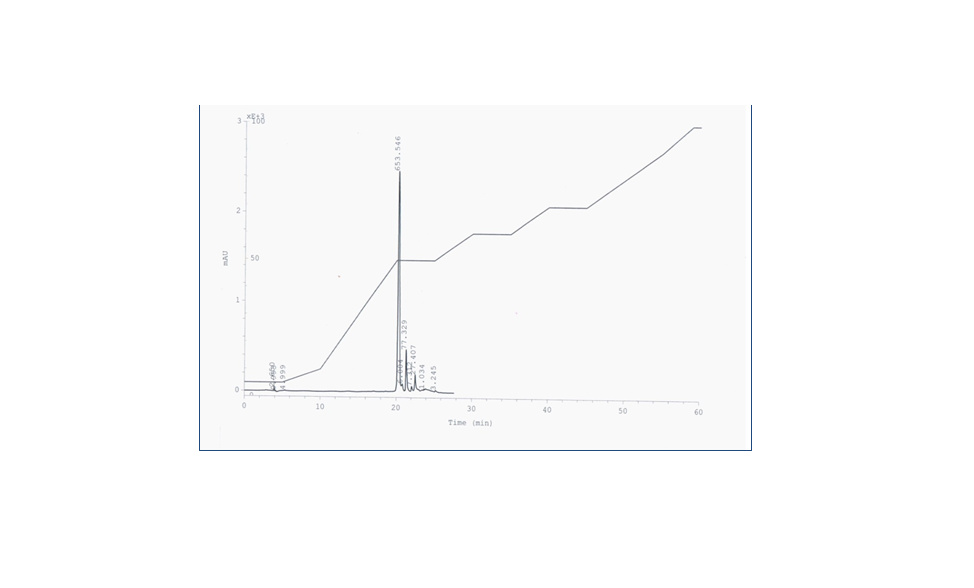

Supplement: S2 Fig — (TIF) [file pone.0202362.s002.tif]

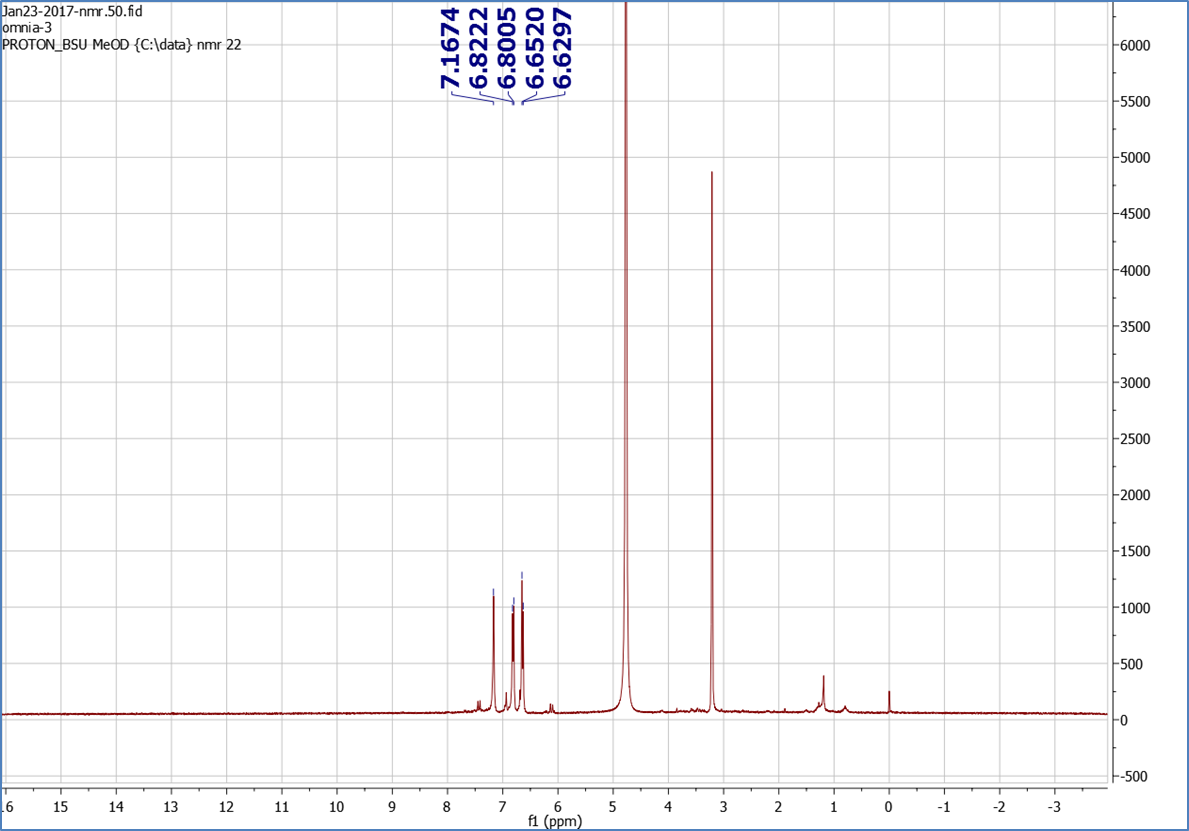

Supplement: S3 Fig — (TIF) [file pone.0202362.s003.tif]

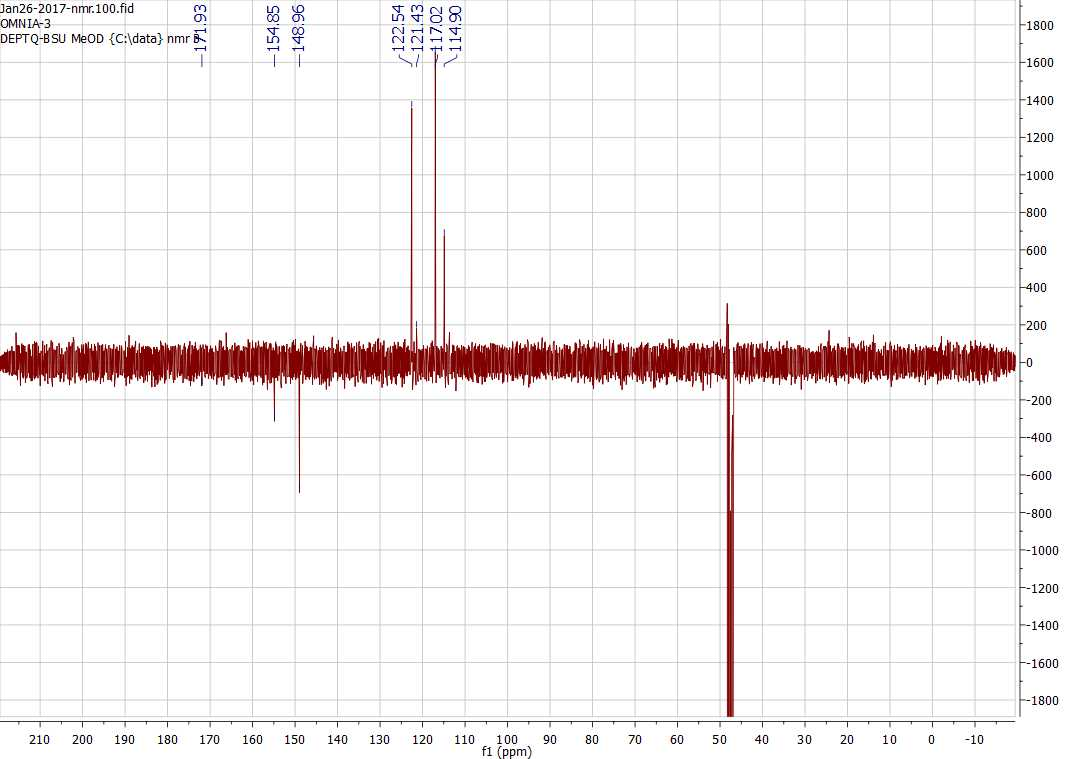

Supplement: S4 Fig — (TIF) [file pone.0202362.s004.tif]

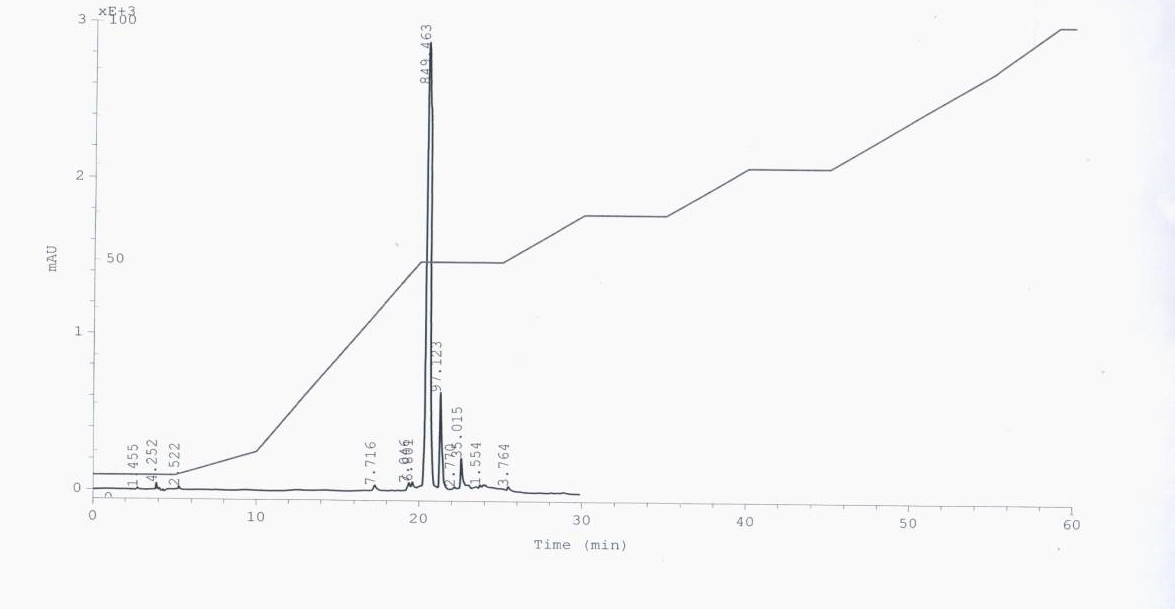

Supplement: S5 Fig — (TIF) [file pone.0202362.s005.tif]

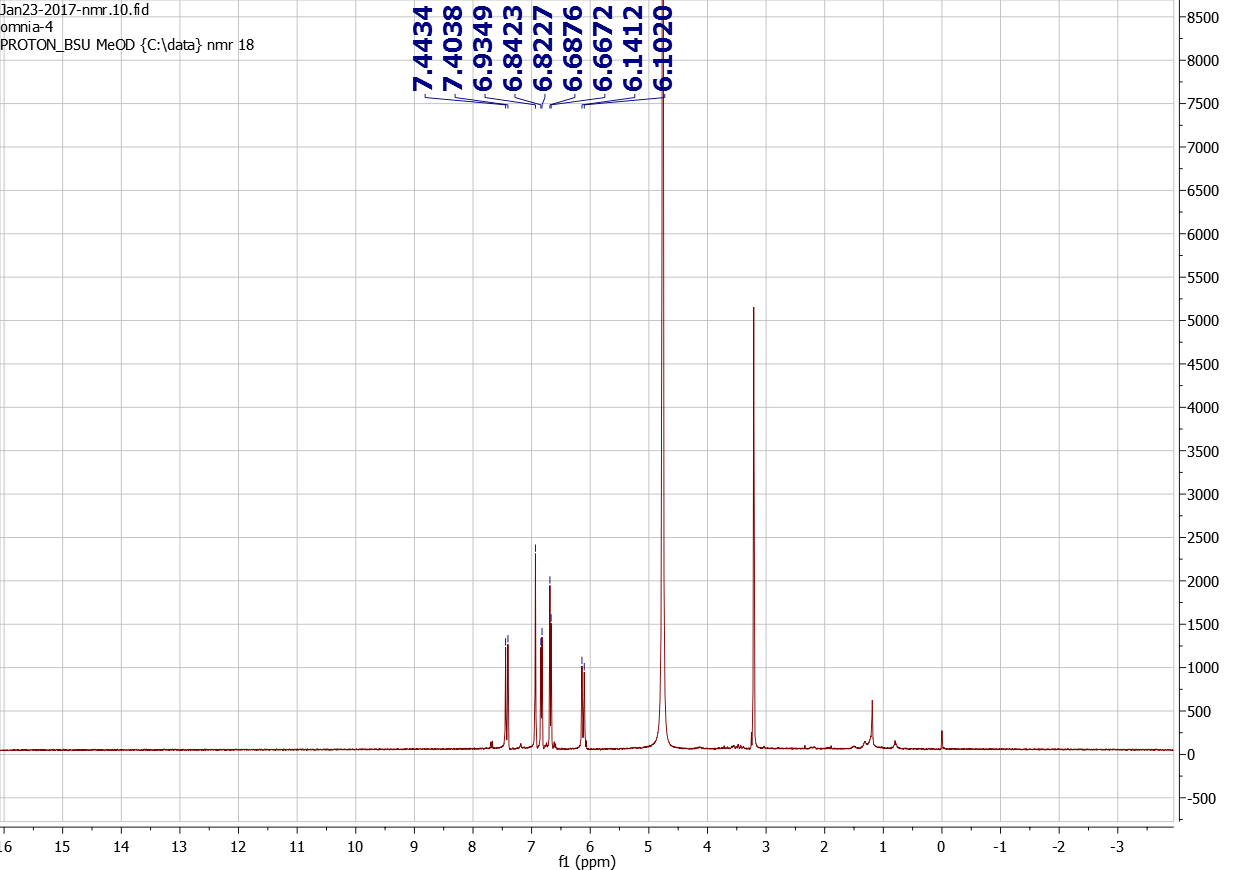

Supplement: S6 Fig — (TIF) [file pone.0202362.s006.tif]

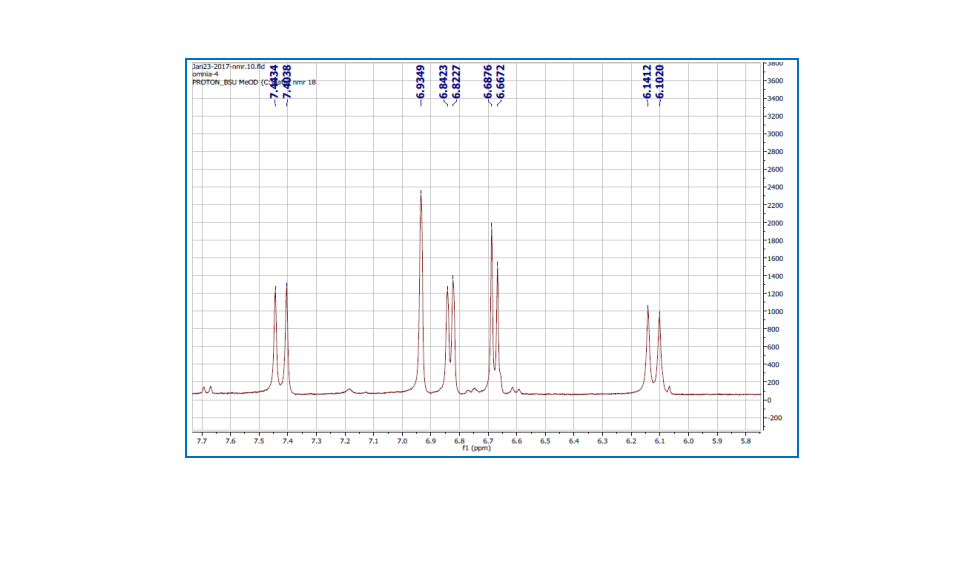

Supplement: S7 Fig — (TIF) [file pone.0202362.s007.tif]

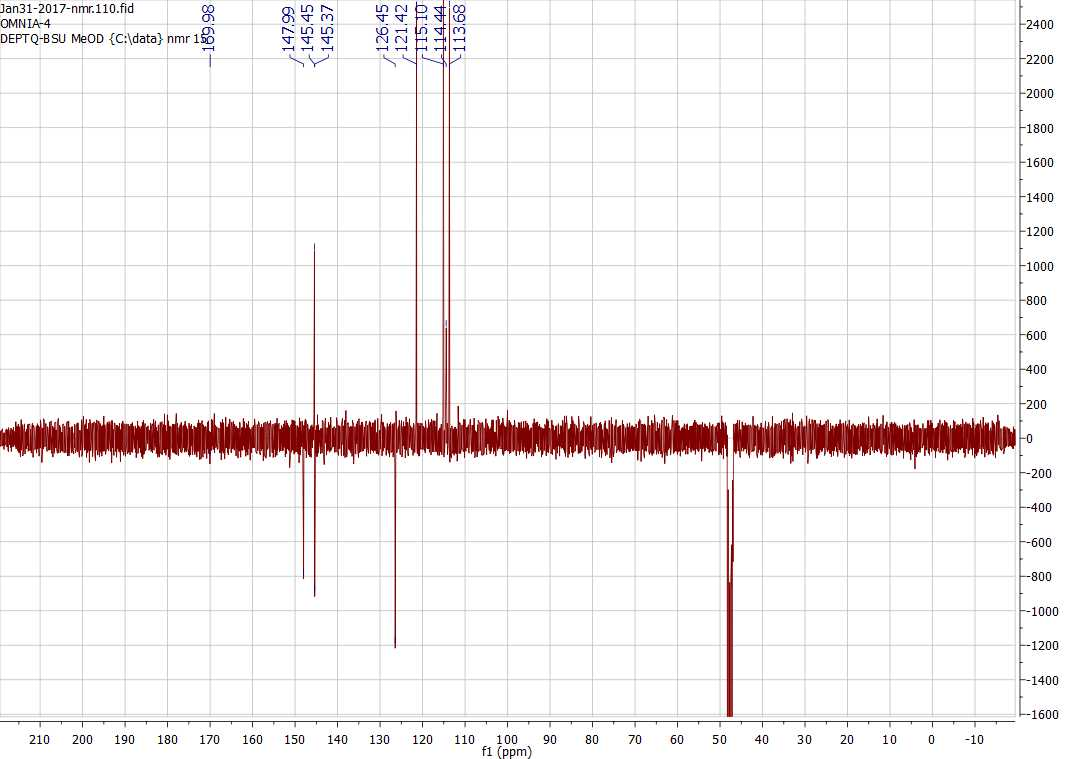

Supplement: S8 Fig — (TIF) [file pone.0202362.s008.tif]

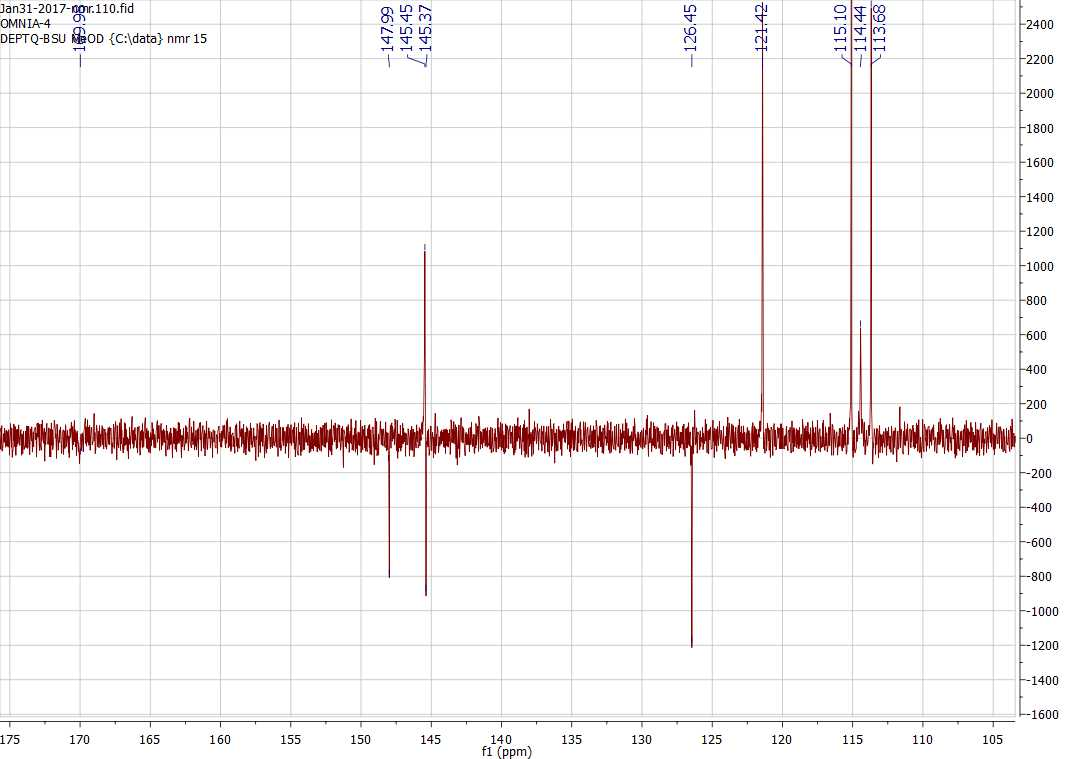

Supplement: S9 Fig — (TIF) [file pone.0202362.s009.tif]

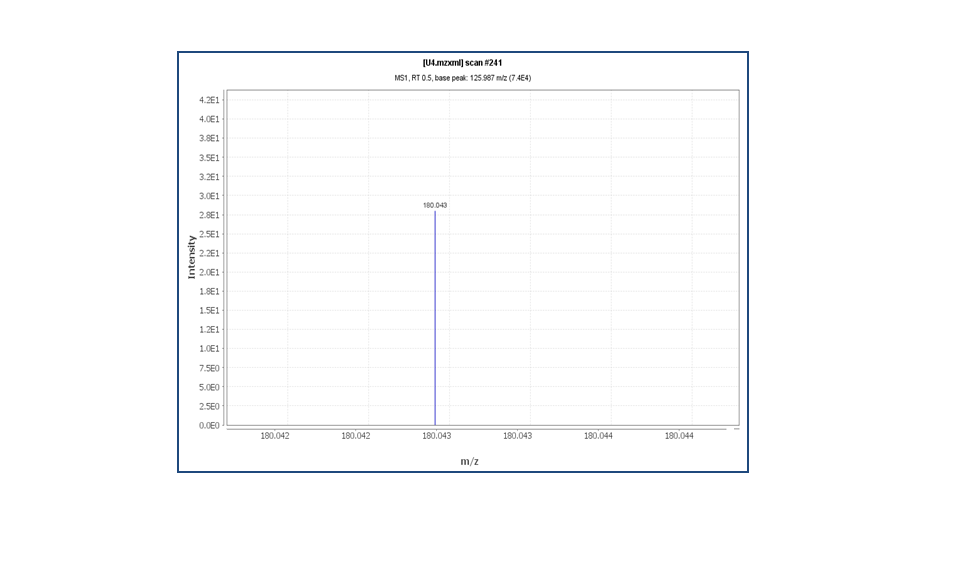

Supplement: S10 Fig — (TIF) [file pone.0202362.s010.tif]

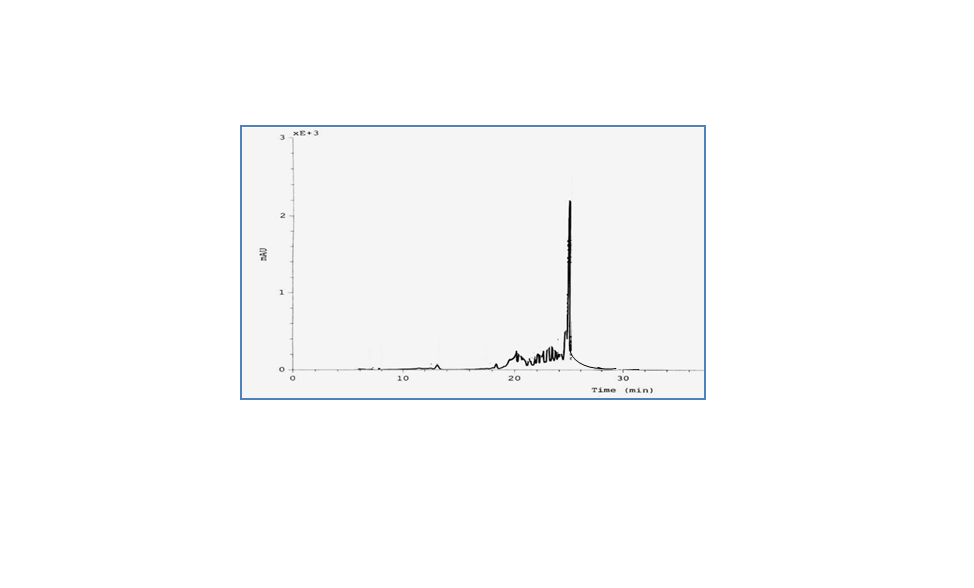

Supplement: S11 Fig — (TIF) [file pone.0202362.s011.tif]

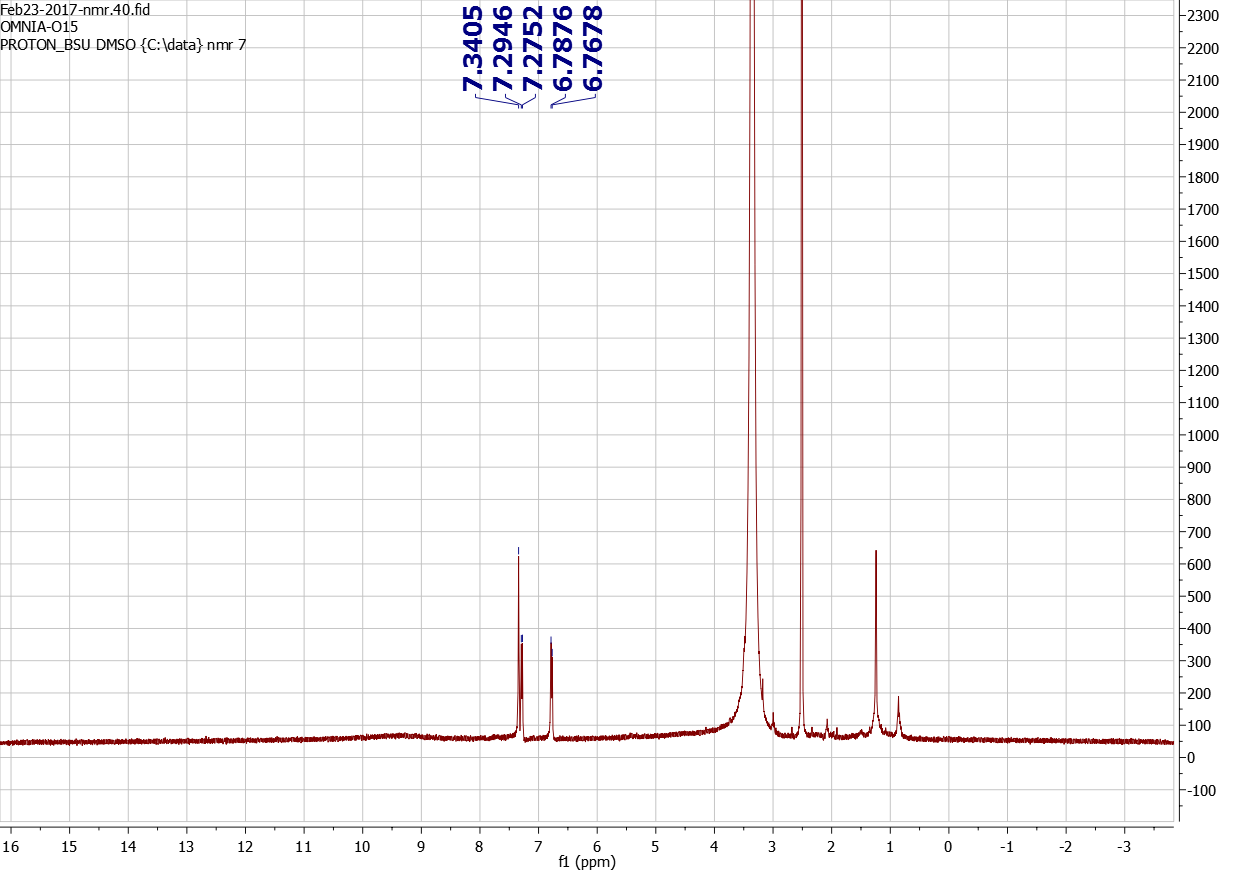

Supplement: S12 Fig — (TIF) [file pone.0202362.s012.tif]

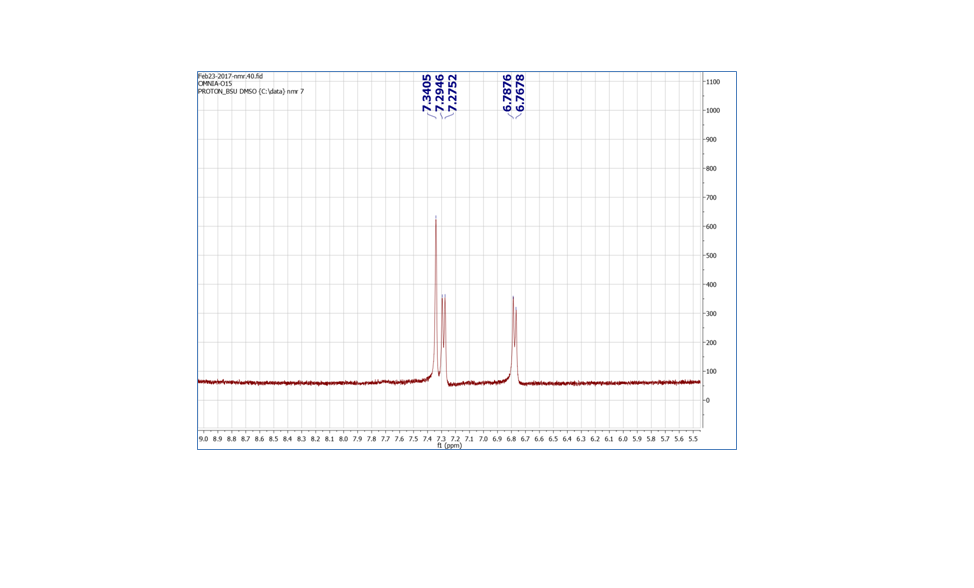

Supplement: S13 Fig — (TIF) [file pone.0202362.s013.tif]

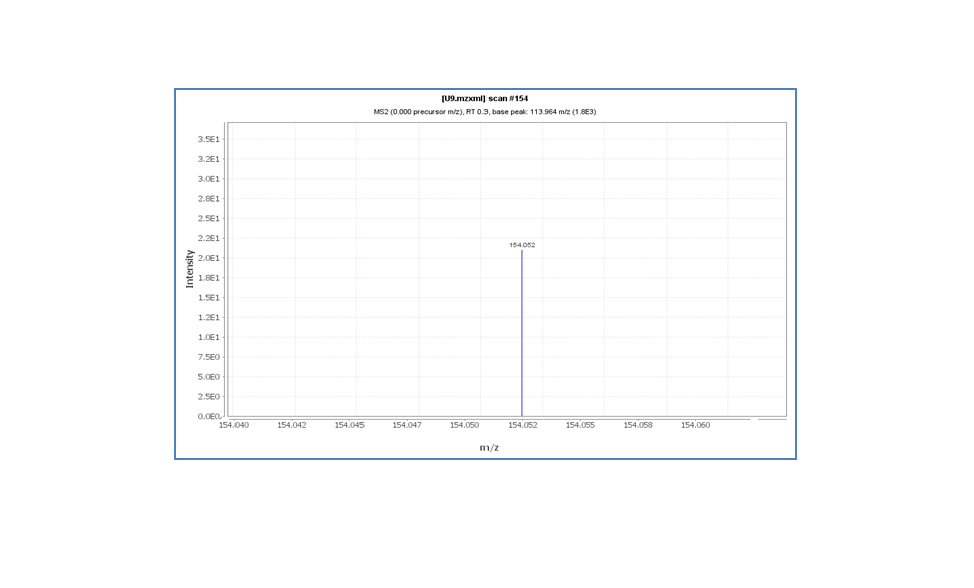

Supplement: S14 Fig — (TIF) [file pone.0202362.s014.tif]

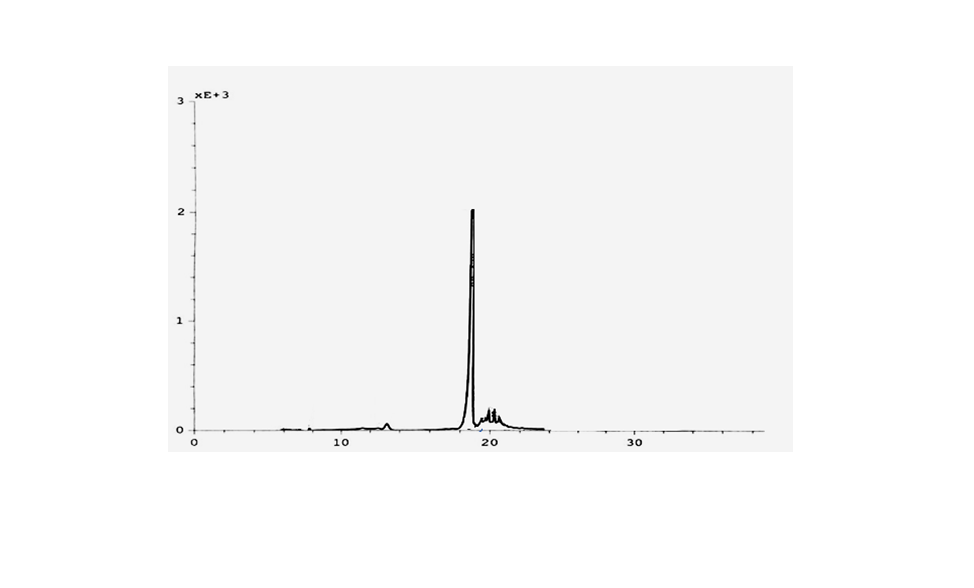

Supplement: S15 Fig — (TIF) [file pone.0202362.s015.tif]

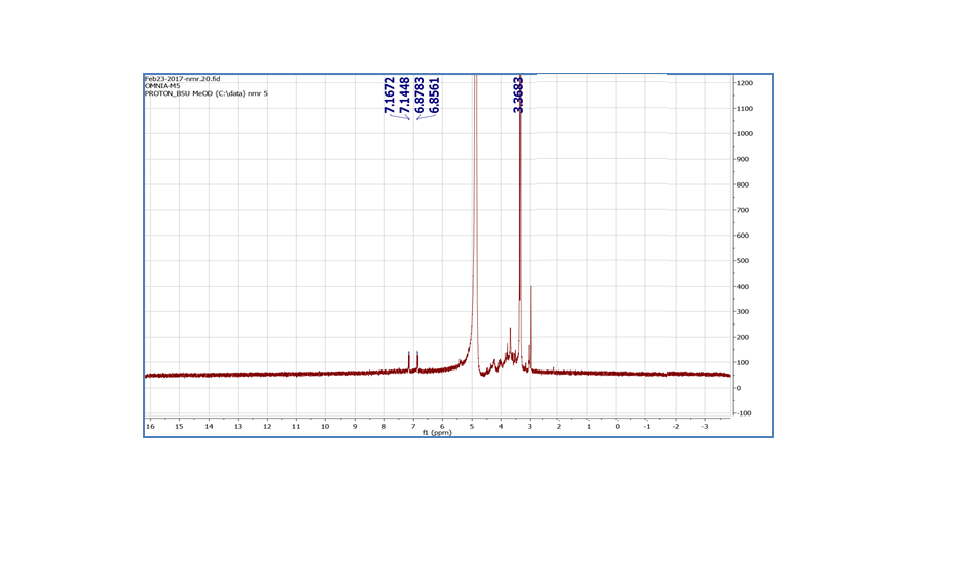

Supplement: S16 Fig — (TIF) [file pone.0202362.s016.tif]

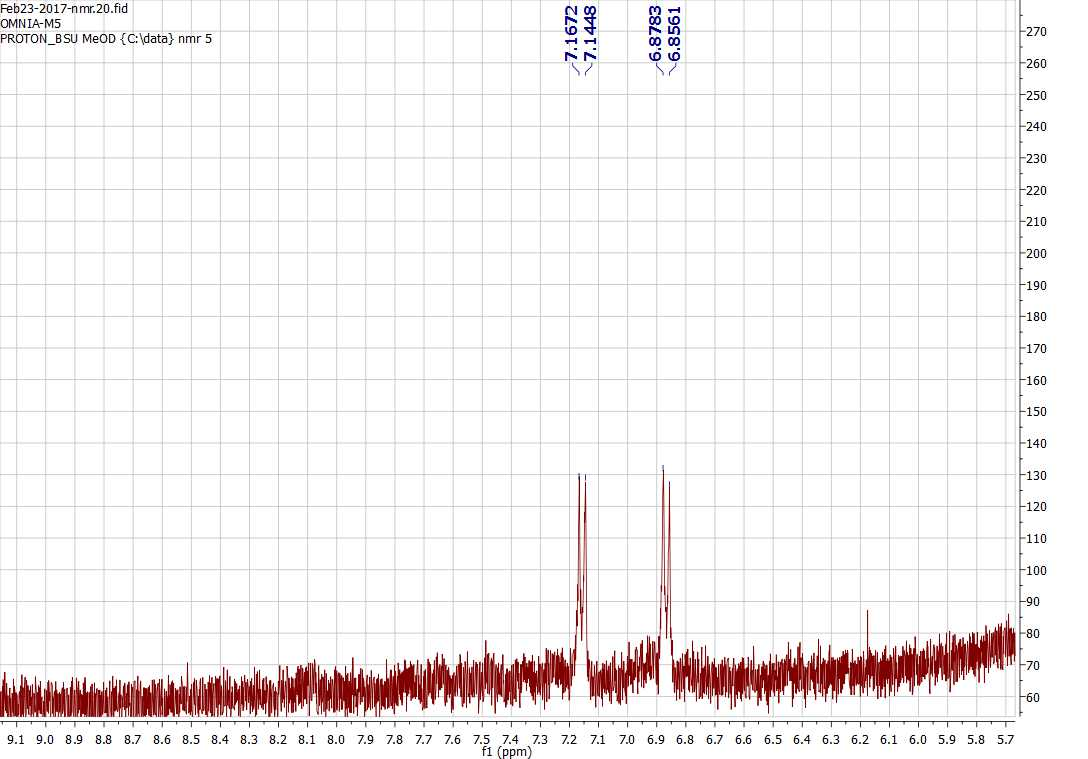

Supplement: S17 Fig — (TIF) [file pone.0202362.s017.tif]

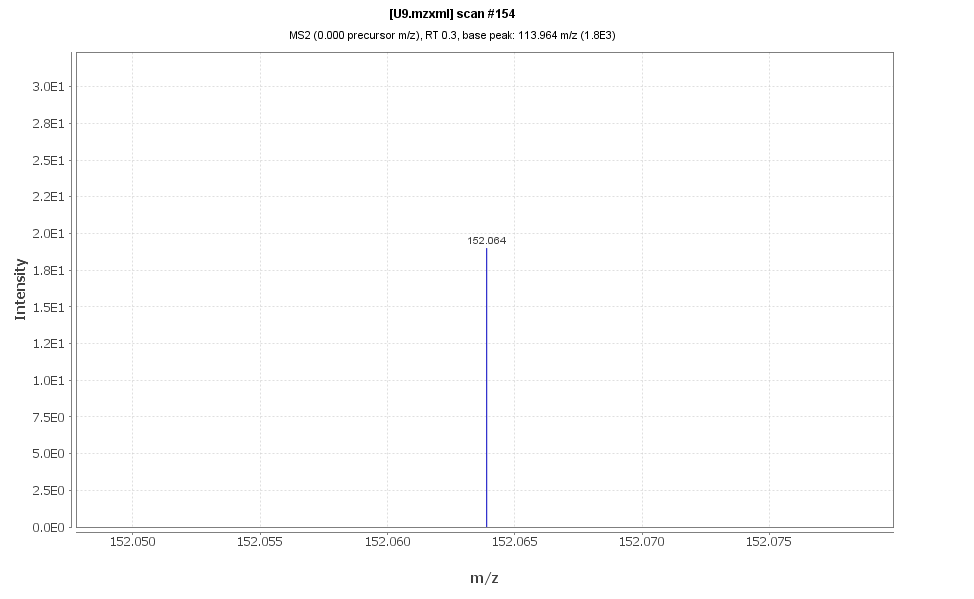

Supplement: S18 Fig — (TIF) [file pone.0202362.s018.tif]

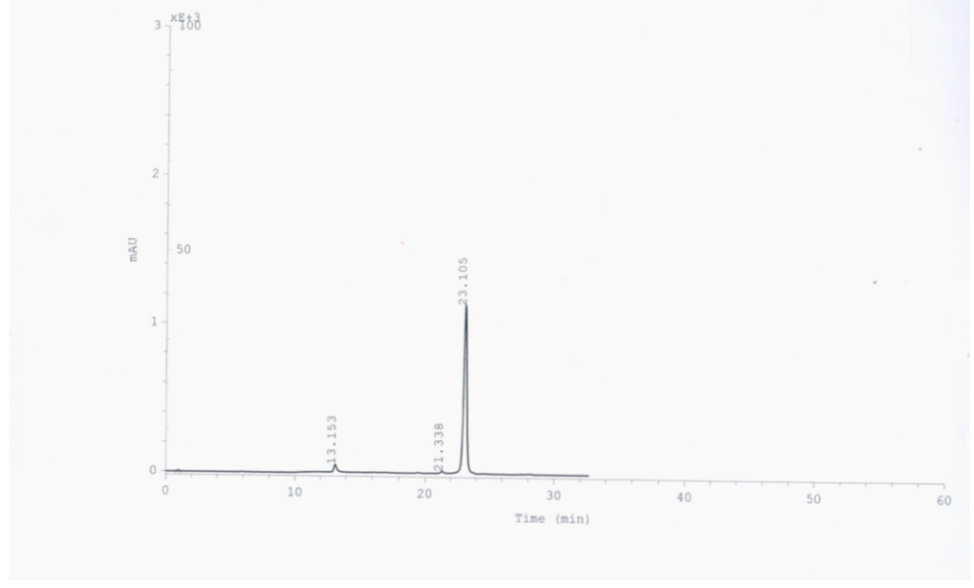

Supplement: S19 Fig — (TIF) [file pone.0202362.s019.tif]

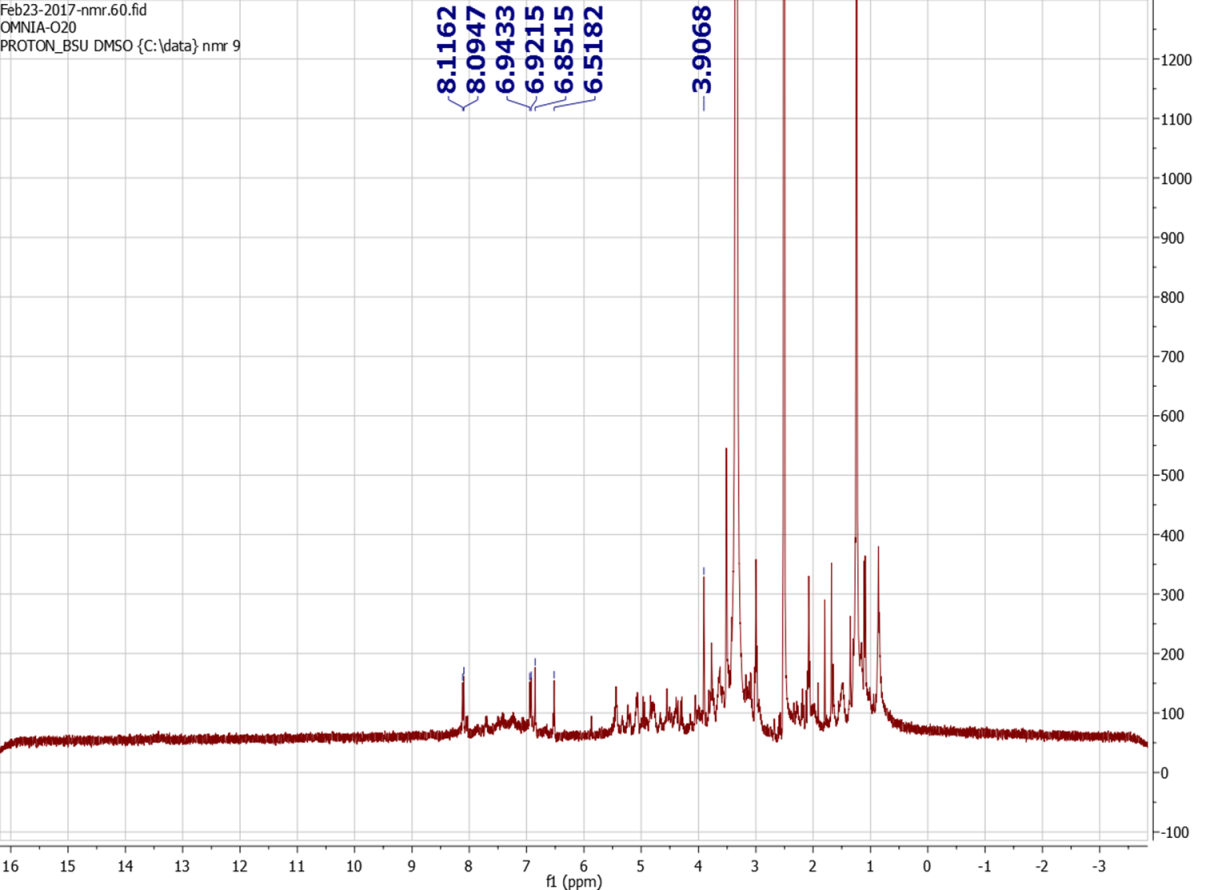

Supplement: S20 Fig — (TIF) [file pone.0202362.s020.tif]

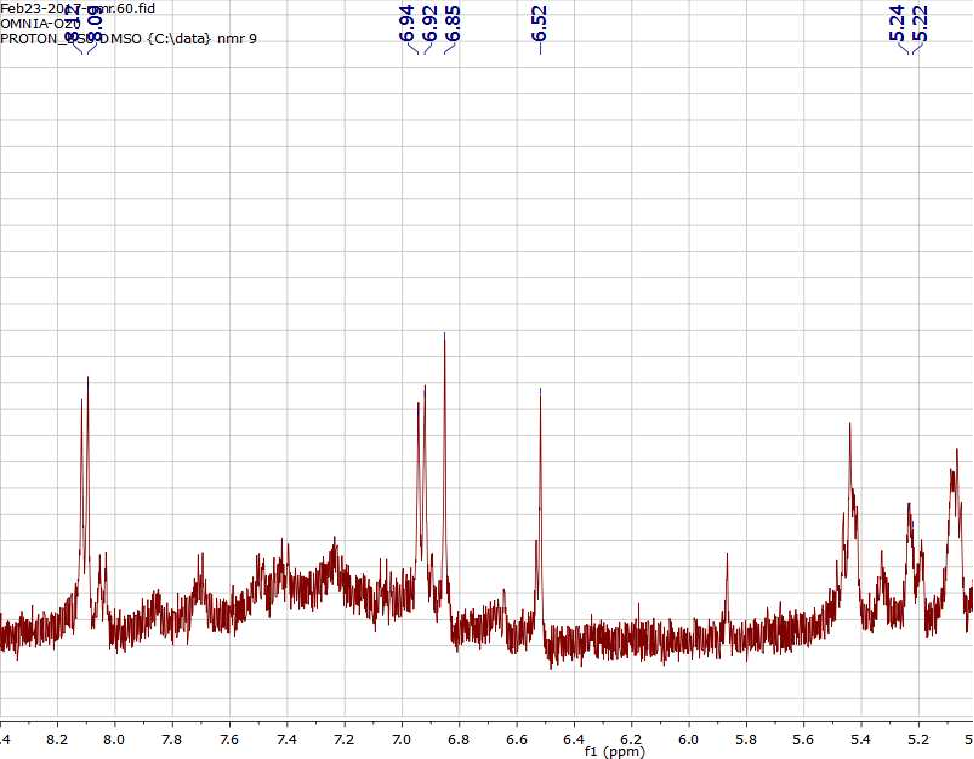

Supplement: S21 Fig — (TIF) [file pone.0202362.s021.tif]

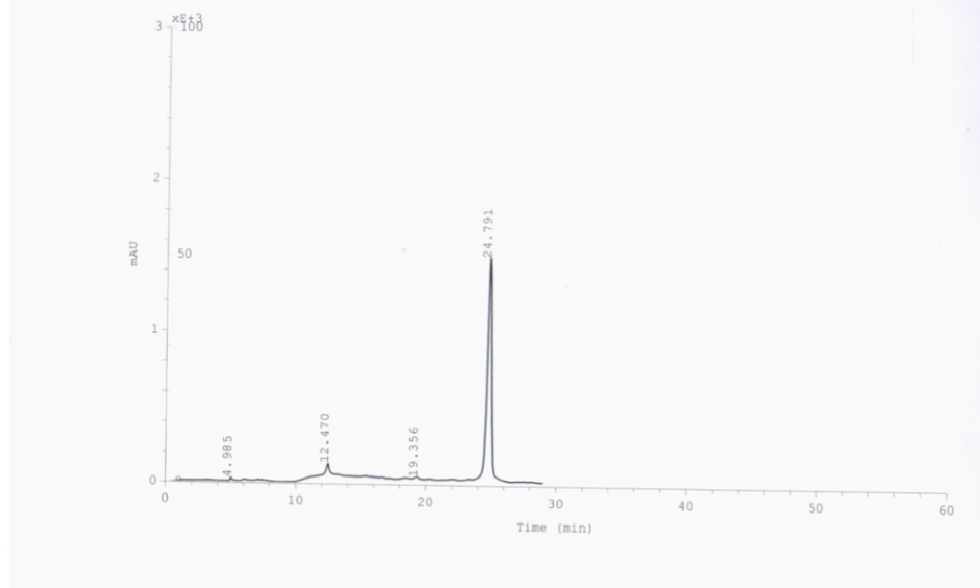

Supplement: S22 Fig — (TIF) [file pone.0202362.s022.tif]

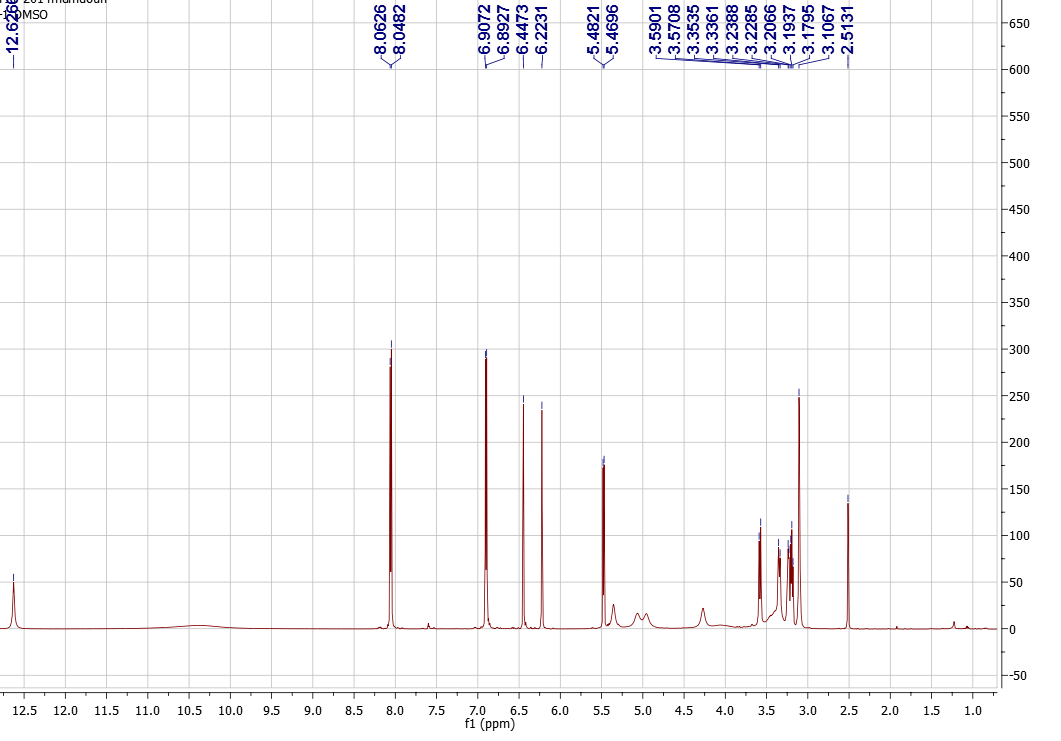

Supplement: S23 Fig — (TIF) [file pone.0202362.s023.tif]

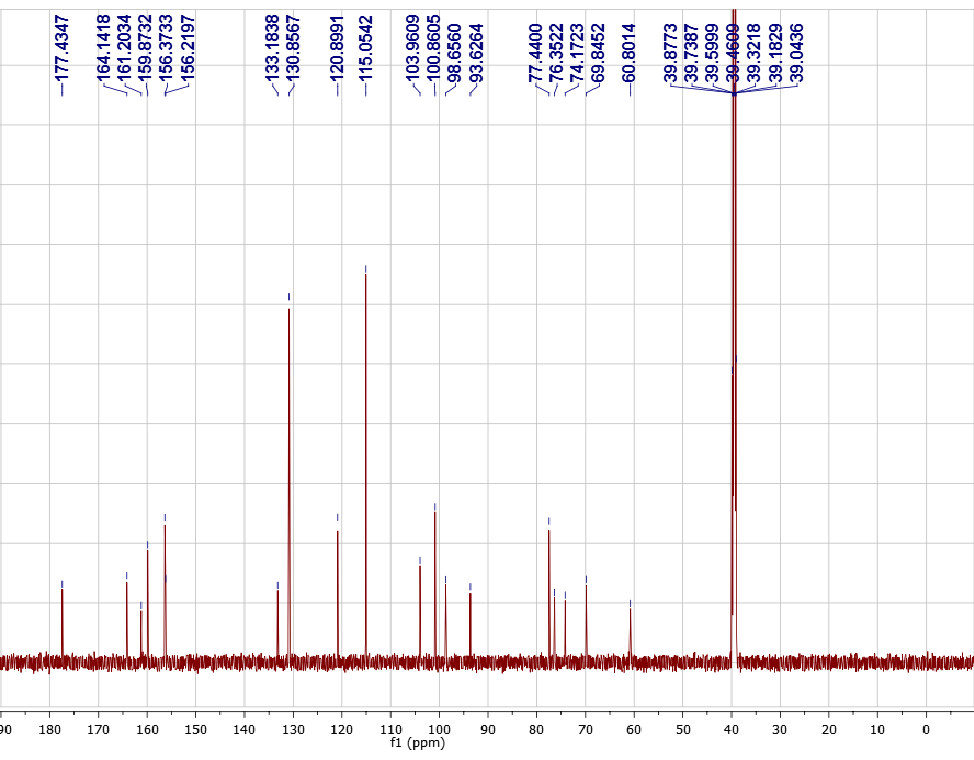

Supplement: S24 Fig — (TIF) [file pone.0202362.s024.tif]
