# Supplementary material for: Hepatoprotective potential of Malvaviscus arboreus against carbon tetrachloride-induced liver injury in rats
Source: PLoS One. 2018 Aug 23;13(8):e0202362. doi: 10.1371/journal.pone.0202362 (PMC6107176; doi:10.1371/journal.pone.0202362)
Supplement: S1 Table — (PDF) [file pone.0202362.s025.pdf]

S1 Table. Effect of the total extract and various fractions of *M. arboreus* on different biochemical parameters in CCl_4-intoxicated rats.

**Test sample/Standard**	**ALT (IU/L)**	**AST (IU/L)**	**ALP (IU/L)**	**TB (mg/dL)**	**MDA (n mole/mg)**	**TAC (n mole/mg)**
Group 1 (Normal control)	49.33 ± 4.10	98.00 ± 6.25	146.70 ± 3.48	0.43 ± 0.06	4.53 ± 0.35	1.90 ± 0.20
Group 2 (CCl_4)	209.70 ± 2.60***	246.00 ± 19.05**	300.70 ± 3.76***	2.03 ± 0.18**	9.05 ± 0.08***	0.91 ± 0.01**
Group 3 (Silymarine + CCl_4)	96.00 ± 9.81***	136.70 ± 7.80**	190.70 ± 7.80***	0.67 ± 0.03**	5.20 ± 0.17***	1.61 ± 0.02***
Group 4 (Pet. ether fraction + CCl_4)	206.00 ± 3.47	213.00 ± 8.09	277.00 ± 15.60**	1.54 ± 0.06	8.15 ± 0.14**	1.10 ± 0.04*
Group 5 (DCM fraction + CCl_4)	96.67 ± 7.22***	136.00 ± 1.16**	204.00 ± 15.01**	0.90 ± 0.04**	6.04 ± 0.08***	1.65 ± 0.02***
Group 6 (EtOAc fraction + CCl_4)	87.67 ± 6.41***	139.70 ± 4.33**	185.00 ± 16.17**	0.68 ± 0.05**	4.73 ± 0.12***	1.49 ± 0.02***
Group 7 (Aqueous fraction + CCl_4)	145.70 ± 4.91**	163.70 ± 2.60*	222.70 ± 4.91***	1.16 ± 0.02**	7.04 ± 0.12***	1.43 ± 0.03***
Group 8 (Total extract + CCl_4)	172.00 ± 9.81*	186.00 ± 6.93*	260.70 ± 3.76**	1.29 ± 0.02*	7.39 ± 0.30**	1.33 ± 0.08**

Values are mean ± S.E.M (n= 6)

* Statistically significant differences ($p < 0.05$, compared with the CCl_4-treated group).

** Statistically significant differences ($p < 0.01$, compared with the CCl_4-treated group).

*** Statistically significant differences ($p < 0.001$, compared with the CCl_4-treated group).
